# An ensemble approach to accurately detect somatic mutations using SomaticSeq

**DOI:** 10.1186/s13059-015-0758-2

**Published:** 2015-09-17

**Authors:** Li Tai Fang, Pegah Tootoonchi Afshar, Aparna Chhibber, Marghoob Mohiyuddin, Yu Fan, John C. Mu, Greg Gibeling, Sharon Barr, Narges Bani Asadi, Mark B. Gerstein, Daniel C. Koboldt, Wenyi Wang, Wing H. Wong, Hugo YK Lam

**Affiliations:** Bina Technologies, Roche Sequencing, Redwood City, 94065 CA USA; Department of Electrical Engineering, Stanford University, Stanford, 94305 CA USA; Department of Bioinformatics and Computational Biology, The University of Texas MD Anderson Cancer Center, Houston, 77030 TX USA; Program in Computational Biology and Bioinformatics, Yale University, New Haven, 06520 CT USA; The Genome Institute, Washington University in St Louis, St Louis, 63108 MO USA; Department of Statistics, Stanford University, Stanford, 94305 CA USA; Department of Health Research and Policy, Stanford University, Stanford, 94305 CA USA

## Abstract

SomaticSeq is an accurate somatic mutation detection pipeline implementing a stochastic boosting algorithm to produce highly accurate somatic mutation calls for both single nucleotide variants and small insertions and deletions. The workflow currently incorporates five state-of-the-art somatic mutation callers, and extracts over 70 individual genomic and sequencing features for each candidate site. A training set is provided to an adaptively boosted decision tree learner to create a classifier for predicting mutation statuses. We validate our results with both synthetic and real data. We report that SomaticSeq is able to achieve better overall accuracy than any individual tool incorporated.

**Electronic supplementary material:**

The online version of this article (doi:10.1186/s13059-015-0758-2) contains supplementary material, which is available to authorized users.

## Background

Cancers are diseases of the genome. Somatic single nucleotide variants (SNVs) and small insertions and deletions (indels) are common drivers of carcinogenesis. Therefore, accurately detecting somatic mutations is a key analysis in cancer research. The challenge and complexity of cancer sequencing analysis lie in the heterogeneous nature of tumor samples, in addition to the cross-contamination between tumor and matched normal samples.

A somatic tool that performs well for one tumor may perform poorly for another, as reported in a number of comparative studies [1, 2]. For instance, MuTect is a somatic SNV caller that applies a Bayesian classifier to detect somatic mutations [3]. It is sensitive in detecting low variant allele frequency (VAF) somatic variants. It also incorporates a series of filters to penalize candidate variants that have characteristics corresponding to sequencing artifacts to increase precision. However, MuTect applies severe penalties to somatic variant candidates if the variant reads are also found in the matched normal. While this approach filters out most germline variant false positives, it adversely affects sensitivity in some cancer types where it is not possible to obtain a clean normal sample, e.g., liquid cancers.

SomaticSniper was developed with the aforementioned issue in mind [4]. It applies a Bayesian model to detect genotype change between the normal and tumor tissues, taking into account the prior probability of somatic mutation. Thus, it is far more tolerant of impure normal samples at the expense of calling a lot more germline variants as somatic. It is also less sensitive toward low VAF mutations. Another Bayesian approach is JointSNVMix2, which jointly analyses paired tumor–normal digital allelic count data [5]. It has very high sensitivity in many different settings, but tends to be lower in precision.

A different statistical approach is using Fisher’s exact test (FET) to detect genotype change, such as VarScan2 and VarDict. VarScan2 reads data from both tumor and normal samples simultaneously and classifies sequence variants by somatic status [6]. At high enough sequencing depth, even a slight change in VAF between the normal and tumor may result in statistical significance by FET, thus calling many germline variants as somatic mutations. On the other hand, VarScan2 will not miss clear mutations due to situation-specific filters that may not appropriately apply in all situations. VarDict is specifically designed to detect important but challenging variants that tend to be missed or ignored by other callers. It applies a series of false positive filters to increase precision [7]. It can handle ultra-deep sequencing with depth up to hundreds of thousands, where most algorithms would either fail or perform poorly.

Given the unique characteristics of each algorithm, integrating them is often desirable to ensure mutations are comprehensively captured [8–10]. On the other hand, combining the false positives from all the different algorithms can easily overwhelm the results. Accurately distinguishing true somatic mutations from the false positives is thus essential in accurate interpretation. Simple rule-based filters can often remove the majority of false positives due to sequencing artifacts, e.g., Database of Single-Nucleotide Polymorphisms (dbSNP) sites, extreme strand bias, nearby homopolymers, low mapping quality, proximity to end of reads, proximity to indels, and extremely low or high read depth [4, 6]. However, hard filters also significantly reduce the sensitivity and permanently remove certain mutations from ever being detected due to their locations within the genome. Previously, Kim et al. built a combined caller using logistic regression with a feature-weighted linear stacking (FWLS) model to improve somatic SNV prediction accuracy [11]. The model considers the degree of consensus of three callers in addition to a series of associated features. It calculates a probability value (0≤*P*≤1) for each mutation candidate; however, which cut-off value to choose is not always obvious. Since the study did not perform somatic indel analysis, its performance on non-substitution variants is unclear.

To address these aforementioned problems, we propose SomaticSeq. It implements a machine-learning algorithm that accurately identifies both somatic SNVs and indels from tumor–normal pairs. It maximizes its sensitivity by combining SNV calls from the five previously described algorithms that complement each other, i.e., MuTect, SomaticSniper, VarScan2, JointSNVMix2, and VarDict. It combines somatic indel calls from Indelocator [12], VarScan2, and VarDict. For each mutation call, we generate up to 72 features by SAMtools, HaplotypeCaller, and the callers themselves. We have implemented the Adaptive Boosting model in R using the ada package [13], which constructs a classifier consisting of an ensemble of decision trees from a training set. The classifier is then applied to a target set to yield a probability (*P*) of a true positive for each somatic variant call.

**Fig. 1:**
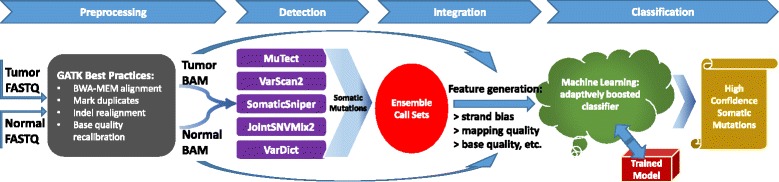
SomaticSeq workflow. The workflow starts with FASTQ files for both the tumor and the matched normal sequencing reads, which are processed using Genome Analysis Toolkit (GATK) best practices to create two BAM files. The five somatic SNV callers (and three indel callers) are run on the pair of BAM files to generate mutation calls. Their results are merged, and then up to 72 features for each of the combined calls are generated from the BAM files using SAMtools and GATK HaplotypeCaller, as well as outputs from the callers themselves. The ensemble along with the feature set is then provided to the machine-learning model, which is trained with either a separate data set or a portion of these data. After training, the model calculates the probability for each call, yielding a high-confidence somatic mutation call set

In this study, we have chosen an optimal *P* (≥0.7) as the probability cut-off value for all of our analyses based on the results from the International Cancer Genome Consortium (ICGC) – The Cancer Genome Atlas (TCGA) Dialogue for Reverse Engineering Assessments and Methods (DREAM) Challenge given in “Results”. One advantage of the stochastic boosting model over FWLS is that the overall accuracy is not sensitive to the choice of *P* over a wide range of values as shown in our results. We have validated SomaticSeq with a variety of synthetic and real tumor data and have achieved high accuracy in the most challenging situations. We use *F*_1_ score, the harmonic mean of precision and sensitivity,

$$F_{1} = 2 \times \frac{\text{sensitivity} \times \text{precision}}{\text{sensitivity} + \text{precision}}, $$

as a measure of overall accuracy. Sensitivity is defined as true positive rate (aka recall) and precision as positive predictive value. A schematic of the SomaticSeq workflow is illustrated in Fig. 1.

## Availability and implementation

The SomaticSeq website [14] is regularly updated with improvements, and also includes links to the source code, releases, and data. The source code for SomaticSeq is deposited and maintained at [15] under a Berkeley Software Distribution (BSD) open-source license. We used SomaticSeq 1.0 [16] for all the analyses in this paper.

SomaticSeq was developed in the Python and Bash scripting languages and can utilize the available hardware parallelism to achieve the best performance. The individual somatic mutation callers are invoked in a region parallel fashion to achieve an efficient run time for the most performance-critical step. Furthermore, SomaticSeq can make use of an available Sun Grid Engine (SGE) cluster to scale performance beyond one hardware node. Since the users may want to run the individual callers with different parameters, SomaticSeq provides the flexibility of configuring the command-line options for these tools. We also emphasize that the machine-learning training component of SomaticSeq has relatively low resource requirements when compared to running the callers. In fact, for the DREAM Challenge data set, which had 30 × coverage, it took around 3 hours to train the classifier and used a maximum of 20 GB of memory. Since training is only done once, this is a one-time cost to incur. The trained classifier is typically 1 GB in size and the prediction step using it only took around an hour per DREAM Challenge data set. Thus, apart from the resource requirements and time to run the individual callers, SomaticSeq incurs little overhead. Finally, the flexibility and performance efficiency make SomaticSeq an extremely useful software resource for cancer researchers. The data used in this study are available via the links in Additional file 1 and at the SomaticSeq website [17].

## Results

We used a variety of real and synthetic data sets to validate SomaticSeq, including the ICGC-TCGA DREAM Somatic Mutation Calling Challenge, in silico titration, SomaticSpike, and real tumor–normal pairs. The data sets used for each procedure are summarized in Additional file 1: Table S1.

### ICGC-TCGA DREAM Somatic Mutation Calling Challenge

First, we present SomaticSeq’s performance on tumor–normal data produced by the ICGC-TCGA DREAM Somatic Mutation Calling Challenge (the DREAM Challenge) [18]. The DREAM Challenge is a community effort to improve bioinformatics algorithms, and in this case somatic mutation detection accuracy. It uses BAMSurgeon to spike mutations computationally into a healthy genome to create synthetic but realistic tumor–normal pairs. The ground truth of the in silico somatic mutations are made public after the challenge deadline. The sequencing depths for both the tumor and normal genomes are approximately 30 ×. Thus, at low VAFs or in low-coverage regions, there may not be any evidence in the data supporting a mutation. While the somatic mutations in these data are synthetic, the rest of the genome is real, containing actual experimental artifacts associated with sequencing technologies and sample preparations that give rise to false positives. The current limitation for using real tumor–normal sequencing data for benchmarking is the lack of ground truth. Thus, the DREAM Challenge is an excellent source of unbiased data for validation.

**Fig. 2:**
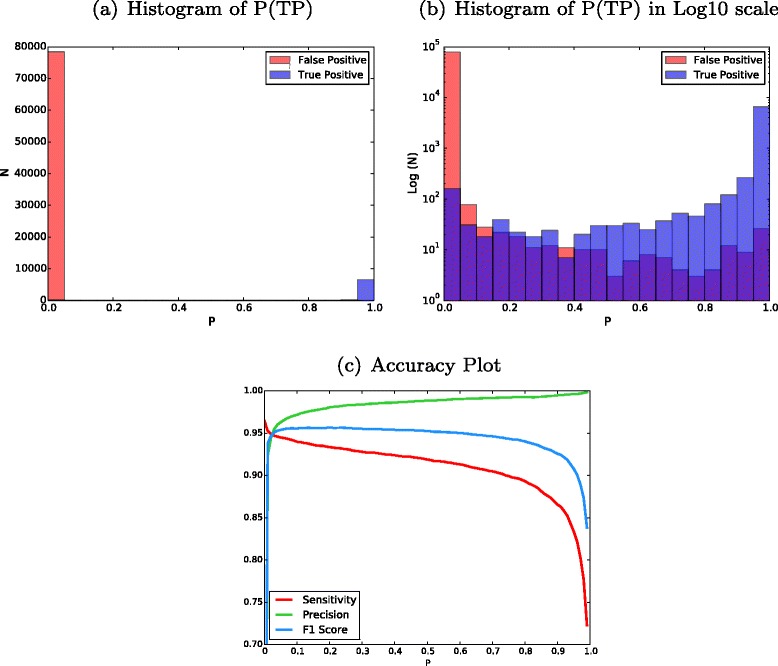
DREAM Challenge Stage 3 results trained from modified Stage 2 data. **a** Histogram of probability values (*P*) of all the mutation candidates in Stage 3. Higher probability values (closer to 1) imply that calls are more likely true somatic mutations. **b** The same plot with the *y*-axis in log10 scale. The overlaps can be seen. Keep in mind each unit in the *y*-axis is a tenfold increase. **c** An accuracy plot showing sensitivity, precision, and *F*_1_ scores vs. *P* cut-off

There are successive stages of the DREAM Challenge, which increase in data complexity, e.g., multiple subclonal populations and simulated contamination to create data sets that are more challenging. The results presented in this study are based on Stage 3, the most complex publishable stage to date. We used a modified data set from a previous stage as the training set for SomaticSeq. Namely, we mixed the tumor and normal data from Stage 2 (no contamination) at 70:30 ratio to create a tumor contamination profile. SomaticSeq found two distinct clusters of calls with different probability values (Fig. 2a, b). The exact accuracy (*F*_1_ score) depended on the choice of cut-off for *P*. Given that the *F*_1_ score was stable over a wide range of *P*, we used a value of 0.7 throughout the study, which slightly favored precision over sensitivity (Fig. 2). Mixing of normal reads into the tumor in Stage 2 data improved the overall accuracy compared to using Stage 2 data directly (data not shown), indicating the importance of having a training set with characteristics similar to those of the target set. The SomaticSeq results presented here were averaged over ten cross-validation results (the training set consists of half of the entire data set, randomly chosen). We performed twofold cross-validation ten times instead of the more common tenfold validation (using 90 % of the data for training) because in some cases choosing 90 % of the data for training would leave too little data for validation. The DREAM Challenge has a permanent website [19], where the data set for each challenge, including the somatic mutations described here, can be downloaded.

**Fig. 3:**
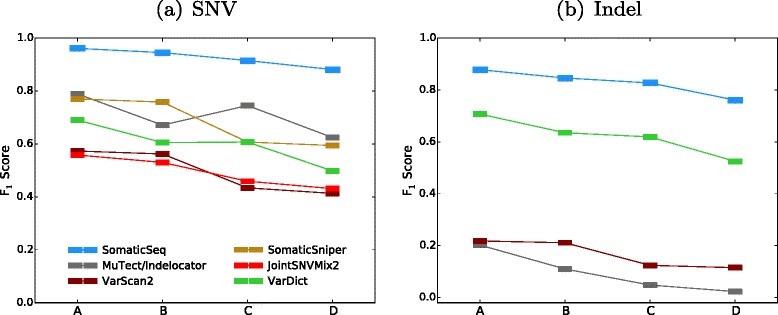
*F*_1_ scores of SomaticSeq and the individual tools for the DREAM Challenge Stage 3 cross-validation. On the x-axes, Setting A is the pure normal/pure tumor. Setting B is the contaminated normal/pure tumor. Setting C is the pure normal/contaminated tumor. Setting D is the contaminated normal/contaminated tumor. **a** SNV results. **b** Indel results

Cross-contamination is a major challenge in real cancer sequencing. Pure tumor or pure normal samples are often impossible to obtain. To evaluate SomaticSeq’s performance in these challenging but more realistic situations better, we created more data sets (Settings) by mixing the tumor and normal data from the DREAM Challenge at different ratios to create different cross-contamination profiles. Setting A was Stage 3 data with no modification, i.e., no contamination, although the tumor had three different VAFs (50 %, 33 %, and 20 %) representing three different subclones. In Setting B, we mixed the normal and tumor data at 95:5 ratio to create a normal sample contaminated with 5 % tumor cells. In Setting C, we mixed the tumor and normal data at a 70:30 ratio to create a tumor contaminated with 30 % normal cells, so the tumor VAFs were 35 %, 23 %, and 14 %. Setting D was the most challenging data set we simulated for the DREAM Challenge, with the normal from Setting B and tumor from Setting C, i.e., the tumor and normal samples were cross-contaminated. The accuracies of SomaticSeq and the individual tools that it incorporates are shown in Fig. 3. SomaticSeq vastly outperforms any individual tool in all situations. For instance, in Setting D, SomaticSeq achieved an *F*_1_ score of 90.5 % (83.2 % sensitivity and 99.4 % precision) in SNVs, whereas the best single tool, MuTect, had an *F*_1_ score of 62.4 % (64.8 % sensitivity and 60.1 % precision). For a more detailed breakdown, see Additional file 1: Table S2.

### In silico titration: accuracy as a function of VAF

**Fig. 4:**
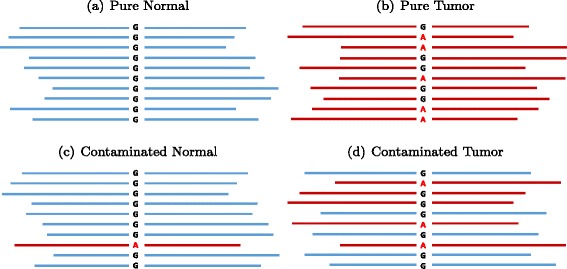
In silico titration of two human genomes. Blue represents reads from NA12878 (designated normal). Red represents reads from NS12911 (designated tumor). Going from (**a**) to (**b**) represents a somatic mutation of G >A, where G in the normal is a homozygous reference and A in the tumor is a heterozygous variant. **c** A normal contaminated with tumor tissues. **d** A tumor sample contaminated with normal tissues

**Fig. 5:**
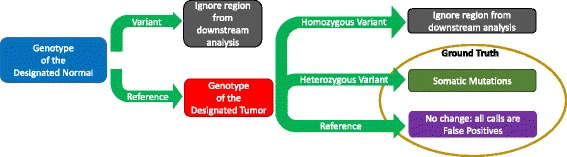
Obtaining the ground truth for the in silico tumor–normal data. In the NA12878 and NS12911 mixture, there are a total of 746,280 virtual somatic SNVs and 64,399 virtual somatic indels. A total of 2.2 billion high-confidence sites are interrogated (the remaining are ignored). During our analyses, a somatic mutation rate of one out of a million was enforced to represent a realistic prior probability of somatic mutations

We performed in silico titration of NA12878 (Platinum genome) and NS12911 (HuRef J Craig Venter genome) to construct partially real tumor–normal pairs of approximately 50 × sequencing depth. We treated NS12911 as the tumor genome and NA12878 as the matched normal (Fig. 4). The ground truth was constructed as illustrated in “Methods” (Fig. 5).

**Fig. 6:**
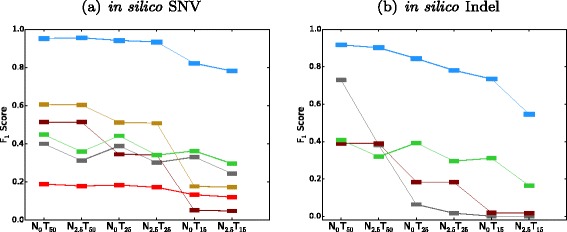
*F*_1_ scores of SomaticSeq and the individual tools in in silico titration. Color legends are shown in Fig. 3. On the x-axes, the subscript denotes the expected VAF as a percentage, i.e., N _0_ *T*_50_ means the normal has VAF = 0 % (i.e., pure normal) and the tumor has VAF = 50 %. N _2.5_ *T*_15_ represents a challenging data set where VAF = 2.5 % for normal and VAF = 15 % for tumor, i.e., 5 % of the normal sample is contaminated with tumor tissues and 30 % of the tumor sample is contaminated with normal tissues. **a** SNV results. **b** Indel results

We mixed the two genomes in silico at different ratios to simulate different cross-contamination profiles, resulting in different VAFs for both the tumor and the normal. Namely, NA12878 and NS12911 were mixed at 0:100, 50:50, and 70:30 ratios to create virtual tumors with target VAFs of 50 %, 25 %, and 15 %. They were also mixed at 100:0 and 95:5 ratios to create a virtual matched normal. Six in silico tumor–normal pairs were created out of those three virtual tumors and two virtual normals. The prior probability of somatic mutation was enforced to be one in a million to make the performance more realistic. SomaticSeq’s performance for these six settings is shown in Fig. 6, and there is a detailed breakdown in Additional file 1: Table S3. The performance of SomaticSeq and each individual tool in the in silico titration was consistent with the results from the DREAM Challenge data. For instance, in Setting N _2.5_*T*_15_, SomaticSeq achieved an *F*_1_ score of 80.5 % (68.4 % sensitivity and 97.8 % precision) in SNVs, whereas the best single tool, VarDict, had an *F*_1_ score of 29.6 % (30.7 % sensitivity and 14.3 % precision).

### SomaticSpike: accuracy as a function of sequencing depth

**Fig. 7:**
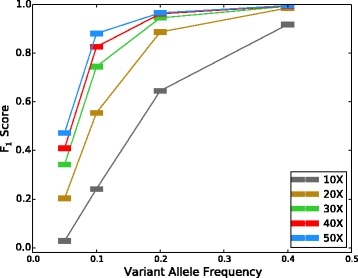
*F*_1_ scores vs. VAF for different coverage depths

SomaticSpike is a method described by Cibulskis et al. for creating virtual tumors with different VAFs and sequencing depths [3]. We used it to test SomaticSeq’s performance. In our SomaticSpike experiment, a pure genome of NA12878 at 30 × was used as the normal sample. To create virtual tumor samples, reads from NA12891 having different genotypes from NA12878 were spiked into the NA12878 genome to create genomes with virtual somatic mutations at those sites. They were spiked at various proportions to create different VAFs. For tumor sequencing depths of 10 ×, 20 ×, 30 ×, 40 ×, and 50 ×, VAFs of 0.05, 0.10, 0.20, and 0.40 were created. The prior probability of somatic mutation was enforced to be one in a million to make the performance more realistic. The overall accuracy (*F*_1_ score) of SomaticSeq was expectedly better with higher sequencing depth and higher VAF (Fig. 7), and outperformed the individual tools that it incorporates (see Additional file 1: Tables S4–S8). For instance, for 50 × sequencing coverage with VAF of 20 %, SomaticSeq achieved an *F*_1_ score of 96.6 % (94.6 % sensitivity and 98.8 % precision), whereas the best single tool, SomaticSniper after having applied the recommended false positive filter, had an *F*_1_ score of 49.0 % (73.6 % sensitivity and 36.7 % precision).

### Real tumor–normal pairs

**Fig. 8:**
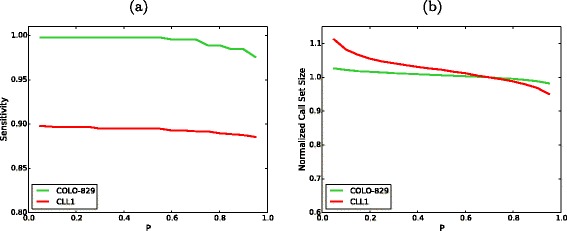
SomaticSeq performance on real data. **a** The sensitivity of SomaticSeq as a function of *P* cut-offs. **b** The call set size as a function of *P* cut-offs, normalized to the call set size at *P*=0.7, i.e., the ratio between the call set size at a given *P* and the call set size at *P*=0.7 (default cut-off in this study)

To demonstrate SomaticSeq’s validity on real sequencing data, we selected two sets of publicly available tumor–normal pairs with published lists of validated somatic mutations as a benchmark for our model. COLO-829 is an immortal metastatic malignant melanoma cell line [20]. The whole-genome sequencing of COLO-829 and its matched normal blood COLO-829BL have sequencing depths of 80 × and 60 ×, respectively. There are 454 validated mutations for COLO-829 [21]. CLL1 is whole-genome sequencing data from a chronic lymphocytic leukemia patient. The sequencing depths for the CLL1 tumor and normal are 53 × and 42 ×, respectively. There are 961 published mutations for CLL1 [22]. Since these data sets only had a subset of known mutations considered as true positives, no information with regard to true negatives (i.e., validated as reference bases) was available. Thus, it was not possible to calculate overall accuracy. In addition, each sequencing center had an analytical pipeline that usually incorporates a popular tool to call somatic mutations. As a result, any validated call was a subset of that particular caller, which would produce 100 % sensitivity if identical settings were used. Due to this limitation, we could not make unbiased comparisons between SomaticSeq and individual callers. Nevertheless, we used the DREAM Challenge Stage 3 data as the training set for SomaticSeq, and obtained sensitivities of 99.6 % and 89.2 % for COLO-829 and CLL1, respectively (Table 1). SomaticSeq’s call set sizes were considerably smaller than those of the individual callers, implying a higher specificity. The call set sizes and sensitivities also varied little with respect to the *P* threshold, consistent with our observation of synthetic data (Fig. 8).

**Table 1:** SomaticSeq sensitivity on real data. ≥*N* tools represents the consensus of at least *N* callers. The two sets of real tumor–normal sequencing data were downloaded from the European Genome Archive. SomaticSeq predictions were trained from the DREAM Stage 3 data sets

Sample	COLO-829		CLL1	
SNV	Number of calls	Sensitivity	Number of calls	Sensitivity
MuTect	46,831	0.996	8,361	0.895
VarScan2	64,927	0.987	19,797	0.888
SomaticSniper	53,077	0.996	13,690	0.907
JointSNVMix2	85,983	0.996	22,534	0.899
VarDict	53,076	0.857	5,748	0.883
Union of five tools	191,696	0.998	55,196	0.935
≥2 tools	57,254	0.996	13,594	0.916
≥3 tools	43,848	0.996	5,836	0.904
≥4 tools	38,216	0.996	2,637	0.886
=5 tools	34,086	0.857	1,749	0.842
SomaticSeq	37,452	0.996	2,320	0.892

**Fig. 9:**
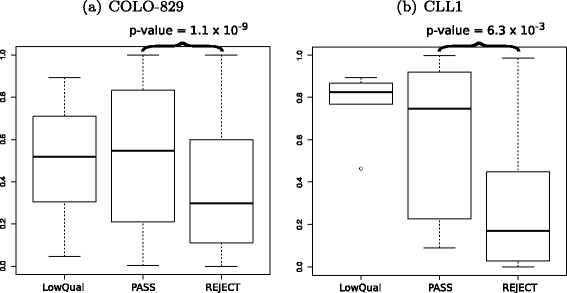
CADD scores from SomaticSeq’s PASS (high confidence) calls vs. LowQual (medium confidence) vs. REJECT (likely false positive) calls. **a** COLO-829. **b** CLL1. Only non-synonymous SNVs were evaluated. The *p*-values were calculated from a two-sided Wilcoxon rank-sum test

To determine if there were any systematic differences in functional annotations of the somatic variants identified as high-confidence mutations (PASS, for *P*≥0.7) vs. likely false positives (REJECT, for *P*≤0.1), the variants were annotated with combined annotation dependent depletion (CADD) scores [23]. SnpEff v4.0 was used to predict the effect of each SNV on the translated protein [24, 25]. We found that likely true somatic mutations (PASS) in both samples were significantly more deleterious than likely false positives (REJECT) by SomaticSeq (Fig. 9). No statistical significance was detected between the PASS and LowQual (for 0.1<*P*<0.7) calls. There were about twice as many PASS calls and 50 times as many REJECT calls as there were LowQual calls. Figure 2b shows that a good number of true somatic mutations can be expected from LowQual calls.

It is reasonable to assume that because cancers are diseases of the genome, deleterious mutations should be enriched in somatic mutations versus random chance. While ground truth was unknown for these data sets, the difference in CADD scores indicated that SomaticSeq’s high-confidence call set was enriched with deleterious variants when compared to the rest of the entire call set.

## Discussion

Each somatic mutation caller implements a unique set of algorithms, with its own assumption of how to discriminate statistically true somatic mutations from sequencing noise. However, each cancer sequencing experiment is different due to the complex nature of different tumor types, and no one algorithm is appropriate for all cancer studies. While clean tumor–normal pairs may be obtained for solid tumors like breast cancer, this may not be feasible for liquid cancers where the samples are far more challenging, such as leukemia or mesothelioma due to expected cross-contamination.

In our modified DREAM data and in silico titration, MuTect performed the best when the normal contained no tumor contamination (see Additional file 1: Tables S2 and S3). It was by far the most sensitive tool among those we have tested when the normal was pure and tumor VAF was very low. On the other hand, it had little tolerance for any contamination in the normal sample, rendering it unsuitable for tumor types where the normal sample is contaminated with tumor cells.

On the other hand, SomaticSniper and VarScan2 look for changes in variant signal between the normal and tumor, at the expense of calling a lot more germline variant false positives. JointSNVMix2, while it over-called mutations more than the other tools in our benchmark, maintained a high sensitivity in a variety of settings, making it a valuable addition to SomaticSeq.

VarDict was the best indel detector when the VAF dipped below 50 %. Additional somatic mutation callers can also be incorporated into SomaticSeq to improve its performance further. However, the value of each additional tool depends on its uniqueness. We aim to incorporate unique algorithms capable of detecting challenging variants missed by others, while the machine-learning model is excellent at discriminating true mutations from false positives in the union of call sets.

A simple consensus approach has been used to improve accuracy [9]. While the accuracies of the consensus approach showed improvement over the individual callers, they were not as accurate as SomaticSeq. In addition, the specific combinations of caller consensus were different depending on the data sets (Additional file 1: Tables S9–S12).

SomaticSeq is also a flexible framework, such that users may choose to run fewer tools due to the limitation of computing resources. In Additional file 1: Tables S13–S15, we show the performance of SomaticSeq incorporating any combination of one to five tools (Additional file 1: Fig. S1). We also show the negative predictive values for these analyses (Additional file 1: Table S16). The accuracies incorporating all five tools are more robust regardless of the data characteristics. Using the right combination of fewer tools in some situations can achieve equally good results, but the optimal combination is not the same for every data set.

Users may also opt to simplify the model by using only the most important features. In Additional file 1: Table S17 and Additional file 1: Fig. S2, we have shown the accuracies of SomaticSeq with the top five, ten, and 20, and full feature sets. The improvement in accuracies is the most pronounced between using only five and ten features, and has diminishing returns as the feature set increases in size. Nevertheless, maximum robustness and accuracy are achieved with the full feature set. We discuss the feature sets in more detail in “Methods”.

We have shown that SomaticSeq is excellent at removing false positives from a call set. However, it relies on the tools we have incorporated to obtain a call set, thus its sensitivity is limited by the individual tools. In its current implementation, all the tools incorporated into the SomaticSeq workflow rely on short read alignment to detect somatic mutation candidates. Thus, it has difficulty detecting mutations that occur in low mappability, ultra-high coverage, low complexity, or otherwise difficult-to-align regions.

For our future work, we may add multi-sample functionality to our approach by incorporating algorithms such as multiSNV, which takes multiple tumor samples from the same patients to take advantage of evolution modeling in heterogeneous cancers to increase the sensitivity and specificity of somatic mutation detection [26]. The tools we have currently implemented assume there is only a single tumor–normal pair. With five such tools incorporated in the workflow, SomaticSeq has been shown to be a highly accurate somatic SNV and indel detector for all types of tumor– normal data.

## Conclusions

SomaticSeq is a flexible, comprehensive, and automated pipeline that incorporates the strengths of different somatic mutation detection algorithms. Components in our pipeline (e.g., aligners, somatic callers, and training features) can be substituted to best suit the needs of the user. It will perform best if the characteristics of the training set are similar to those of the target set. Nevertheless, SomaticSeq provides a default trained model from high-quality synthetic data with which we have demonstrated its high accuracy and robustness.

## Methods

### Somatic mutation callers

While many individual callers have optional input parameters that can improve prediction results for sequencing data of different characteristics (e.g., ploidy, purity, mutation rate, etc.), those parameters are usually unknown to researchers. Thus, in most cases, we used default or recommended settings for each caller. For MuTect, we supplied dbSNP version 138 [27], Catalogue of Somatic Mutations in Cancer (COSMIC) version 69 [28], and a panel of normal based on Phase 1 of the 1000 Genomes Project as resource files for the real sequencing data. We did not supply COSMIC for the DREAM Challenge, because the synthetic mutations were randomly chosen and were not enriched in COSMIC sites. In our in silico titration and SomaticSpike experiments, none of these databases was used.

For SomaticSniper, we used a mapping quality cut-off of 25, a base quality cut-off of 15, and a prior somatic mutation probability of 10 ^−4^. For VarScan2, we used a mapping quality cut-off of 25 and a base quality cut-off of 20. For JointSNVMix2, we used a convergence threshold of 0.01 in training, and only considered calls with a somatic probability ≥0.95. For VarDict, we relaxed some built-in filters to increase its sensitivity (at the expense of precision). Specifically, we relaxed the variant depth filter from 4 to 2, and the FET *p*-value cut-off from 0.05 to 0.15. In addition, we allowed each call to fail for up to two out of 20 VarDict filters.

These parameters were chosen from our experiences with Stage 3 of the DREAM Challenge. Except for the different resource files we have supplied to MuTect, we used the same configuration for all of our analyses presented in this study.

### SomaticSeq workflow

The complete SomaticSeq workflow is illustrated in Fig. 1. From the raw sequencing reads, BAM files for the normal and tumor sequencing reads are generated using GATK best practices [29]. From the tumor–normal BAM files, a union of somatic SNVs is called from MuTect, SomaticSniper, VarScan2, JointSNVMix2, and VarDict. Somatic indels are called from VarScan2, VarDict, and Indelocator (aka SomaticIndelDetector). For each of the mutation candidate positions, we integrate and standardize the feature sets. We use SAMtools [30] and GATK HaplotypeCaller on the tumor and normal BAM files to obtain a number of independent sequencing features that have predictive values for their somatic mutation statuses, e.g., mapping quality, base call quality, strand bias, depth of coverage, tail distance bias, etc. Some caller features, e.g., somatic mutation scores based on its distinct statistics, are also included. For the DREAM Challenge and real data, we also consider whether the site is in dbSNP. Two of the most important features in the adaptively boosted classifiers include the root-mean-square mapping quality score and the number of read mismatches compared to the reference. Histograms visualizing some of the features’ predictive values are shown in Additional file 1: Fig. S3.

We use the R package ada to train the stochastic boosting machine-learning algorithm in SomaticSeq [13]. The stochastic boosting learner constructs a classifier consisting of a sequence of decision trees based on up to 72 genomic and sequencing features to discriminate true somatic mutations in the training set. The constructed classifier is then applied to a target set, and calculates the probability of each candidate site being a true somatic mutation (Fig. 2). Some of those features are stronger predictors than others, but they all add some value to the model. When all features are combined, the model is very accurate. For the results described in this study, we have used *P*≥0.7 as the cut-off for our SomaticSeq results, i.e., a candidate site of *P*≥0.7 is considered a PASS call, whereas a candidate site of *P*<0.7 is considered LowQual. The cut-off value of 0.7 is chosen to prioritize slightly precision over sensitivity, though the actual accuracies tend to be very robust to a wide range of values (Fig. 2).

### Trained models

The importance of each feature differs from data set to data set, but there is a lot of overlap for the most important features. In all settings of the DREAM Challenge SNV data, 18 of the top 20 features overlapped. They are listed as follows (not ranked):

- Classification by MuTect (binary values of 0 or 1)
- Classification by JointSNVMix2
- Classification by SomaticSniper
- Classification by VarDict
- FET somatic *p*-value reported by VarDict
- Average mapping quality in the tumor BAM file
- Average mapping quality in the normal BAM file
- Forward and reverse read counts supporting a variant in tumor
- Forward and reverse read counts supporting a reference in tumor
- Forward and reverse read counts supporting a reference in normal
- Read depth in tumor
- Read depth in normal
- Number of mismatches (compared to reference) in tumor reads
- dbSNP membership (binary values of 0 or 1)
- Strand bias odds ratio reported by VarDict

Four of the top 18 features were simple classifications made by the individual tools (whether or not the caller has called it a somatic mutation). The only caller classification not on the list was VarScan2, although it was outside the top 20 only in Setting D. For Settings A, B, and C, the VarScan2 classification was ranked number 16, 20, and 17, respectively. Because VarScan2 has a series of (tunable) filters prior to evaluations, e.g., a minimum VAF of 10 % and minimum coverage of 8 ×, VarScan2’s sensitivity in challenging data sets like Setting D is reduced. Other important features are related to the quantity of evidence (e.g., read counts and read depth), quality of evidence (read mismatch and mapping qualities), and sequencing artifacts (strand bias). Prior knowledge (dbSNP membership) was also valuable. Since eight of the top 18 features related directly to sequencing depth, it is important for the trained model to have a comparable sequencing depth as the target set. Thus, it would not be appropriate to use a 30 × whole-genome sequence trained model to predict somatic mutations in a 500 × targeted sequencing, e.g., three variant reads in 30 × data imply 10 % VAF, but 3 reads in 500 × data imply 0.6 % VAF, which is very little evidence over expected sequencing errors.

A trained model consists of an ensemble of decision trees with different relative weights. For the model trained from the combined DREAM Challenge Settings A and B, which we used to predict real data, the top decision tree can be described as follows:



T_MQ is the root-mean-square mapping quality in the tumor BAM file. VarScan2_Score is the Phred-scale FET *p*-value reported by VarScan2. The asterisks denote terminal nodes. This is the number 1 decision tree from the trained model we used to predict somatic mutations for the publicly available data (Table 1). The tree view is presented in Additional file 1: Fig. S4. This trained model, along with the indel model trained from the same data set, can be downloaded from our Git repository [14].

**Fig. 10:**
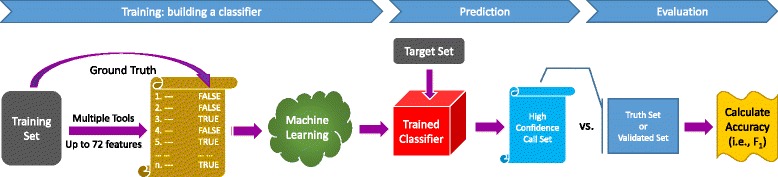
Machine learning: a training set with ground truth is provided to the machine-learning algorithm to create an adaptively boosted classifier. The classifier is applied to a target set to create a high-confidence somatic mutation call set. The call set is compared to the ground truth or the validated mutation list of the target set to calculate the accuracy (only sensitivity is calculated for real data)

The prediction accuracy of the target set largely depends on the similarity between the training and target sets. Thus, ideally, a randomly sampled subset of the same data is used for training as described by Kim et al., e.g., randomly choose 500–1000 mutation candidates from a large sequencing study for validation sequencing, to construct a training set with hundreds of true somatic mutations and confirmed true negatives [11]. It is important to keep the training and target sets similar in terms of sequencing depth, platforms, and identical data processing. For instance, if different mappers are used, or if quality scores are calibrated differently, then the predictions may be less accurate. In addition, it may not be appropriate to use a carcinogen-driven tumor like lung cancer to predict somatic mutations in pediatric cancer, because the mutation profiles of those two types of cancers are vastly different. We have tested the performance of SomaticSeq with a small subset of the data (i.e., a random sampling that includes 10–1000 true somatic mutations), and have presented the results in Additional file 1: Tables S18 and S19, and Additional file 1: Figs. S5 and S6, for somatic SNVs and indels, respectively. Unsurprisingly, the accuracy improves with increasing number of data points in the training set, with diminishing returns after the training set reaches around 100–200 true somatic mutations. However, improvements over individual tools are shown with just 10–20 true mutations in the training set. However, in the absence of such a training set, a synthetic data set with characteristics close enough to the target set would also suffice, as we have done for COLO-829 and CLL1 with the DREAM Challenge Stage 3 for training. In this study, we calculate the accuracy of SomaticSeq’s predictions based on the known ground truth for synthetic data (i.e., DREAM Challenge, in silico titration, and SomaticSpike) and published lists of mutations for real data (Fig. 10).

### In silico titration

We mixed two human genomes (NA12878 and NS12911) in silico to create a virtual tumor–normal sequencing experiment as illustrated in Fig. 4. To create any mixture of the two genomes, the two BAM files were randomly downsampled at the appropriate fraction, and then merged together using Picard tools. To construct the ground truth from the in silico experiment, if a location is a homozygous reference in NA12878 (designated normal) and heterozygous in NS12911 (designated tumor), this location was considered to be a somatic mutation. If the site is a homozygous reference in both genomes, it was considered a reference, so any somatic call that fell in those regions was considered a false positive. All other genotyping possibilities were considered ambiguous for somatic analysis, and were ignored in the downstream analysis. The schematic to build the ground truth is illustrated in Fig. 5. One major difference between our in silico titration and SomaticSpike is that we used two entirely different genomes as the tumor and normal, whereas SomaticSpike used the same genome for both, but selectively spiked in alternate reads from a different genome as virtual mutations. Our in silico titration was not only able to capture sequencing artifacts associated with sequencers, but it also captured artifacts associated with two separate sample preparations as would be expected from some tumor and normal studies.

To obtain the highest confidence truth set, heterozygous variant calls in NS12911 must be agreed upon by three germline callers: HaplotypeCaller, FreeBayes, and SAMtools. In addition, we only considered the high-confidence callable regions (minimum depth of 10, minimum mapping quality of 20, and minimum base quality of 10) in both NA12878 and NA12911 for this exercise.

On average, there is at least one single-nucleotide polymorphism per 1000 bp in a human genome, and this in silico titration created over 810,000 virtual somatic mutations, which presented a prior somatic mutation rate of about one out of 2700. This was orders of magnitude more frequent than the “rule of thumb” of one somatic mutation out of every million base pairs [31]. Thus, using all 810,000 virtual somatic mutations would be a poor evaluator of a somatic caller’s precision because true hits would unrealistically outnumber false positives. It was necessary to enforce a realistic somatic mutation rate to evaluate better the precision of each tool in real cancer analysis. Therefore, 2218 somatic SNVs and indels were randomly chosen, while the remainder were masked from our analysis. In SomaticSpike, where we investigated SomaticSeq’s performance as a function of sequencing depth and VAF, we randomly chose 3000 somatic SNVs to enforce a one in a million prior mutation probability. We also refrained from using dbSNP or COSMIC membership as features for the in silico experiments, because their membership statuses in these databases did not reflect the reality in real cancers. In other words, the results for the in silico titration presented in this study did not take information from any prior knowledge.

In in silico titration, we created impure tumors by mixing tumor and normal sequencing reads. Some of the reads in the virtual impure tumor were identical to reads in the normal, without expected experimental artifacts because the normal contamination in this case did not come from normal tissues, but directly from the normal data. This led to an inflated precision since identical reads cannot “fool” the caller. Therefore, to obtain a realistic number of false positives, we used the false positives obtained from pure normal/pure tumor analysis as false positives for all in silico analyses. In SomaticSpike, a separate tumor/normal whole genome analysis was done for every sequencing depth, and false positives from that were used for every VAF study of that sequencing depth.

### Functional annotation

SNVs reported by SomaticSeq were annotated with CADD rank scores in dbNSFP v2.8, where the scores were provided for every non-synonymous SNV. Rank score is a ratio of the rank of the raw score over the total number of raw scores in dbNSFP. Larger values indicate relatively higher deleteriousness. The Wilcoxon rank-sum test was conducted using pairwise.wilcox.test in R with multiple testing corrections using the Holm–Bonferroni method (see Fig. 9). All SNVs reported by SomaticSeq were also annotated with raw CADD scores generated using CADD v1.2 and the raw scores were converted to rank scores (see Additional file 1: Fig. S7).

## Additional file

Additional file 1
**Supplementary text.** It contains detailed results of our analyses in **Figures S1–S7** and **Tables S1–S19.** It also describes the location of the data used in this paper. (PDF 531 kb)

## Abbreviations

BSD: Berkeley Software Distribution
CADD: combined annotation dependent depletion
COSMIC: Catalogue of Somatic Mutations in Cancer
dbSNP: Database of Single-Nucleotide Polymorphisms
DREAM: Dialogue for Reverse Engineering Assessments and Methods
FET: Fisher’s exact test
FWLS: feature-weighted linear stacking
GATK: Genome Analysis Toolkit
ICGC: International Cancer Genome Consortium
indel: small insertion/deletion
SNV: single nucleotide variant
TCGA: The Cancer Genome Atlas
VAF: variant allele frequency
