# Supplementary material for: An ensemble approach to accurately detect somatic mutations using SomaticSeq
Source: Genome Biol. 2015 Sep 17;16(1):197. doi: 10.1186/s13059-015-0758-2 (PMC4574535; doi:10.1186/s13059-015-0758-2)
Supplement: Additional file 1 — Supplementary text. It contains detailed results of our analyses in Figures S1–S7 and Tables S1–S19. It also describes the location of the data used in this paper. (PDF 531 kb) [file 13059_2015_758_MOESM1_ESM.pdf]

1. Detailed SomaticSeq Results

Table 1 summarizes what training data were used for each study in the paper.

1.1. DREAM Challenge. The cross validation results for DREAM Challenge are presented in Table 2. We also investigated SomaticSeq's accuracy in challenging regions (e.g., regions of low mappability, low complexity, etc). DREAM Challenge simulated only 25 mutations in regions ENCODE considered to be unmappable (in the wgEncodeDacMapabilityConsensusExcludable.bed file from UCSC). 24 out of those 25 mutations were captured by the combined five callers, and a total of 2,939 false positives were called. Thus, before any filtering, the union of 5 call sets had a F_1 score of 1.61% (recall of 96.0% and precision of 0.81%). After 10 cross-validations by SomaticSeq, the F_1 scores for DREAM Challenge's settings A, B, C, and D were 96.7%, 94.4%, 94.0%, and 84.3%. Their recalls were 93.6%, 94.4%, 93.6%, and 77.6%. Their precisions were 100%, 94.4%, 94.4%, and 92.4%. These results were comparable to rest of the data sets in Table 2, but keep in mind that only 25 mutations were simulated in those regions, and did not necessarily represent the reality. In general, SomaticSeq is not expected to perform too well in low mappability regions in its current implementation, because all the callers it has incorporated depend on short read mapping as a precursor to variant calling.

1.2. *in silico* titration. The cross validation results for *in silico* titration are presented in Table 3. SomaticSeq* (one asterisk) results in Table 3 were obtained as follows: for each of the 6 *in silico* settings, we randomly split them into two halves. We combined one half of them into a single data set for training. Then, we used that trained classifier to predict mutation status for each of the other halves of the 6 *in silico* settings. We ensured that the training and target data did not overlap. We then reversed the training/testing in the other direction, and averaged the two results. The results from this method were equally good or better than cross validation. This was likely due to the fact that, with 6 times the amount of training data in this method versus cross validation, a more accurate trained model was built. The extra data from the different *in silico* settings differed only in the number of variant reads, and this small discrepancy in the number of variant reads did not disrupt the accuracy of the trained model.

SomaticSeq** (two asterisks) results in Table 3 were trained from the combined DREAM Settings A and B. Keep in mind that the DREAM Challenge and *in silico* titration data were a gross mismatch in terms of sequencing characteristics. DREAM Challenge contained synthetic mutations, where the tumor and normal reads came from a single experiment (reads randomly split into the designated tumor and normal, with synthetic mutation spiked into the tumor). The *in silico* titration's tumor and normal came from two different human genomes sequenced at two different sequencing centers. The results were less accurate than cross validation, but still substantively more accurate than any individual tool. This implies that the DREAM Challenge data is sufficiently realistic, that the model trained from DREAM Challenge substantially improved prediction results over any individual tool.

1.3. SomaticSpike titration. The detailed cross validation results for SomaticSpike are presented in Tables 4 to 8.

2. Additional SomaticSeq Analyses

2.1. Simple consensus. A simple consensus is to take the intersections of multiple callers (i.e., mutation calls agreed upon by multiple tools), and label calls with at least N number of tools as high confidence calls. This simple approach improved accuracy over individual callers as well, but paled in comparison with SomaticSeq, and was less robust over different types of data. The F_1 score, sensitivity, and precision for every combination of somatic SNV call consensus are summarized in Tables 9, 10, and 11. Tables 12 shows the sensitivity of the real data with this consensus approach.

2.2. Reduced number of tools incorporated. The bulk of the computing resources used in SomaticSeq is running the individual tools it has incorporated. While the addition of each tool adds to our combined sensitivity, there are enough features in our model so that our precisions are not overly dependent on any of the tools. Fig. 1 shows how the F_1 score scales with the number of tools for SNVs. The gain in accuracy is more pronounced in the most challenging data sets, but the addition of each tool shows diminishing return. Tables 13, 14, and 15 detail the F_1 score, recall, and precision of SomaticSeq incorporating every possible combination of tools.

2.2.1. *Negative predictive values (NPV).* Negative predictive value (NPV) is the fraction of true negative calls over all negative calls. Since the rate of somatic mutation is typically around 1 out of a million, NPV over the whole genome is close to 1 for all sensible somatic mutation callers. However, we calculated our NPV not over the entire genome, but over the union of call sets. This evaluates SomaticSeq's ability to filter out false positives from the call set. Results are shown in Table 16.

2.3. **Reduced feature sets.** SomaticSeq used up to 72 features to discriminate true somatic mutations from the data sets. All features have some predictive values, but some have much greater predictive values than others. We have already listed the top 18 features in the Method section of the main text, and also tested the algorithm with only the top 5, 10, and 20 features (Table 17). The prediction accuracies improve with more features, but diminish after about 20 most valuable features (Fig. 2). The top 5 features were strand bias odd ratio, normal read depth, tumor mapping quality, MuTect classification, and variant reverse read counts (should be the same weight as forward read counts, but in choosing top 5, only one made the cut). The next 5 top features rounding up the top 10 were variant forward read counts, VarDict classification, VarDict's somatic score, normal mapping quality, and JointSNVMix2 classification. Fig. 3 visualizes the breakdown of true somatic mutations vs. false positives for some features of different importance in the Stage 3 of DREAM Challenge.

The classifier from a trained model is an ensemble of decision trees with different relative weights. The number of decision trees is the number of iterations during training. Decision trees in the beginning are more heavily weighted than decision trees at the end. The decision tree shown in the main manuscript is also represented as Fig. 4. if_MuTect, if_VarDict, if_JointSNVMix2 are classifications by these tools, and hold binary values of 0 or 1. if_dbsnp represents membership in dbSNP, and also holds binary values of 0 and 1. VarScan2_Score is the Phred-scaled Fisher's exact test p-value reported by VarScan2. SomaticSeq has in essence reclassified VarScan2's calls in this tree to have a more strict p-value cut off. T_MQ is tumor mapping quality which ranges from 0 to 60 for BWA aligned reads.

Some features were turned off in certain cases, e.g., all features related to dbSNP were turned off for *in silico* titration and SomaticSpike analysis, because most virtual somatic mutations in those two data sets were in dbSNP, but in reality mutation candidates in dbSNP tend to be germline variant false positives. Features related to SomaticSniper and JointSNVMix2 were effectively turned off for INDEL analyses because these two tools do not call INDELs. Indel length was effectively turned off for SNV because it's always zero.

2.4. **Reduced size of training sets.** Tables 18 and 19 (Fig. 5 and 6) shows SomaticSeq accuracy as a function of the size of training set. The prediction accuracy improved with increasing size, but reached diminishing return when there were about 200 true positives in the training set. A call set typically has more false positive than true positives, thus we recommend the size of training set should be large enough to include at least 100 true positives. If in rare cases when the number of true positives outnumbers false positives, the size of the training set should include at least 100 false positives instead.

3. Data Availability

For the DREAM Somatic Mutation Challenge data, GeneTorrent is required to download the BAM files. GeneTorrent is available at UCSC: `https://cghub.ucsc.edu/software/downloads.html`. The public key for DREAM Challenge is located at `http://dream.annailabs.com/dream_public.pem`. Specifically, we have used Stage 2 and Stage 3 data from DREAM Challenge in this paper. The URL's of the data for GeneTorrent are:

- Stage 2 Normal:
  `https://dream.annailabs.com/cghub/data/analysis/download/865fa3d6-2024-47cf-bf66-c258f8c0efcf`
- Stage 2 Tumor:
  `https://dream.annailabs.com/cghub/data/analysis/download/7cf8416e-6055-4a0e-86ee-b719db9fbc16`
- Stage 3 Normal:
  `https://dream.annailabs.com/cghub/data/analysis/download/b19d76a0-a487-4c50-8f9c-3b4d5e53239d`

Test Set	Training Set	Validation Method
Original DREAM Challenge Stage 3	Modified DREAM Challenge Stage 2	Compare to ground truth
Modified DREAM Challenge Stage 3	Half of the Test Data	Cross validation with ground truth
in silico Titration and Somatic-Spike	Half of the Test Data	Cross validation with constructed truth set
COLO-829	Original DREAM Challenge Stage 3	Compare to experimentally validated mutations
CLL1	Original DREAM Challenge Stage 3	Compare to experimentally validated mutations

TABLE 1. Specifying what training data are used for each test data set in this study, and what data is used for validation.

- Stage 3 Tumor:
  `https://dream.annailabs.com/cghub/data/analysis/download/8fe6fc33-2daf-4393-929f-7c3493d04bef`

For our *in silico* titration, the two genomes can be downloaded at the following locations:

- NA12878 (virtual normal):
  `ftp://ftp.sra.ebi.ac.uk/vol1/fastq/ERR194/ERR194147`
- NS12911 (virtual tumor):
  `http://www.ncbi.nlm.nih.gov/sra/SRX1016818`

For SomaticSpike:

- NA12878 (virtual normal):
  `ftp://ftp-trace.ncbi.nih.gov/1000genomes/ftp/technical/working/20120117_ceu_trio_b37_decoy/`. The file names are `CEUTrio.HiSeq.WGS.jaffe.b37_decoy.NA12878.chr*.clean.dedup.recal.20120117.bam`.
- NA12891 (virtual tumor):
  `ftp://ftp-trace.ncbi.nih.gov/1000genomes/ftp/technical/working/20120117_ceu_trio_b37_decoy/`. The file name is `CEUTrio.HiSeq.WGS.b37_decoy.NA12891.clean.dedup.recal.20120117.bam`.

For the real data, they are downloaded from European Genome Archive (EGA).

- CLL1:
  `https://www.ebi.ac.uk/ega/datasets/EGAD00001000023`
- COLO829:
  `https://www.ebi.ac.uk/ega/studies/EGAS00000000052`

DREAM	Recall				Precision				F_1 Score			
SNV	A	B	C	D	A	B	C	D	A	B	C	D
MuTect	0.931	0.721	0.849	0.648	0.684	0.627	0.664	0.601	0.789	0.671	0.745	0.624
VarScan	0.711	0.689	0.503	0.468	0.245	0.239	0.186	0.176	0.364	0.354	0.272	0.255
VarScan+Filter	0.666	0.648	0.459	0.432	0.502	0.496	0.411	0.396	0.573	0.562	0.434	0.413
SomaticSniper	0.776	0.755	0.542	0.524	0.493	0.486	0.404	0.396	0.603	0.591	0.463	0.451
Sniper+Filter	0.746	0.726	0.519	0.503	0.797	0.792	0.731	0.725	0.770	0.758	0.607	0.594
JointSNVMix	0.899	0.839	0.692	0.638	0.404	0.388	0.343	0.325	0.558	0.530	0.459	0.431
VarDict	0.883	0.726	0.731	0.555	0.567	0.518	0.520	0.451	0.690	0.605	0.607	0.498
Union of Tools	0.961	0.942	0.892	0.851	0.138	0.135	0.129	0.124	0.241	0.237	0.226	0.216
SomaticSeq	0.937	0.923	0.875	0.832	0.992	0.997	0.996	0.994	0.964	0.958	0.932	0.905
INDEL												
Indelocator	0.170	0.088	0.037	0.018	0.248	0.146	0.067	0.033	0.202	0.110	0.048	0.023
VarScan	0.375	0.361	0.202	0.186	0.154	0.149	0.089	0.083	0.218	0.211	0.124	0.115
VarScan+Filter	0.127	0.123	0.069	0.065	0.238	0.233	0.146	0.139	0.165	0.161	0.094	0.089
VarDict	0.753	0.640	0.616	0.490	0.667	0.630	0.621	0.565	0.707	0.635	0.619	0.525
Union of Tools	0.847	0.791	0.762	0.667	0.212	0.201	0.195	0.175	0.339	0.320	0.310	0.277
SomaticSeq	0.807	0.776	0.754	0.656	0.952	0.985	0.986	0.985	0.874	0.868	0.855	0.788

TABLE 2. VarScan+Filter and Sniper+Filter contains a subset of calls where the author-recommended false positive filters are applied to the original call sets. Setting A: Stage 3 data straight up. Setting B: the matched normal is contaminated with 5% tumor. Setting C: the tumor is contaminated with 30% normal, in which case variant allele frequencies of 35%, 23%, and 14% are present in the tumor sample. Setting D: combination of C and D, i.e., the normal is contaminated with 5% tumor, and tumor is contaminated with 30% normal. The ensemble contained all calls from VarScan2 and SomaticSniper (without false positive filter), plus with VarDict's internal filters relaxed.

TYPE	Recall						Precision						F_1 Score					
VAF_T	50%		25%		15%		50%		25%		15%		50%		25%		15%	
VAF_N	0%	2.5%	0%	2.5%	0%	2.5%	0%	2.5%	0%	2.5%	0%	2.5%	0%	2.5%	0%	2.5%	0%	2.5%
MuTect	0.991	0.734	0.957	0.706	0.787	0.551	0.250	0.198	0.244	0.192	0.210	0.157	0.400	0.312	0.389	0.302	0.331	0.244
VarScan	1.000	1.000	0.606	0.599	0.078	0.070	0.170	0.170	0.111	0.109	0.016	0.014	0.291	0.291	0.187	0.185	0.026	0.024
VarScan + Filter	0.896	0.896	0.541	0.534	0.071	0.064	0.360	0.360	0.253	0.251	0.043	0.039	0.514	0.514	0.345	0.342	0.053	0.048
SomaticSniper	1.000	0.992	0.791	0.783	0.219	0.212	0.208	0.207	0.172	0.171	0.054	0.053	0.344	0.342	0.283	0.280	0.087	0.084
Sniper + Filter	0.892	0.885	0.705	0.698	0.199	0.193	0.460	0.458	0.402	0.400	0.160	0.156	0.607	0.604	0.512	0.508	0.177	0.173
JointSNVMix	1.000	0.943	0.968	0.904	0.680	0.619	0.104	0.099	0.101	0.095	0.073	0.067	0.189	0.179	0.184	0.172	0.133	0.121
VarDict	0.819	0.620	0.805	0.583	0.629	0.307	0.308	0.252	0.305	0.241	0.255	0.143	0.448	0.359	0.442	0.341	0.363	0.296
Union of Tools	1.000	1.000	0.995	0.985	0.917	0.830	0.061	0.061	0.061	0.060	0.056	0.051	0.116	0.116	0.115	0.114	0.106	0.097
SomaticSeq	0.947	0.936	0.917	0.903	0.721	0.684	0.963	0.969	0.978	0.985	0.959	0.978	0.955	0.952	0.946	0.942	0.823	0.805
SomaticSeq*	0.915	0.947	0.917	0.932	0.698	0.703	1.000	1.000	1.000	1.000	1.000	1.000	0.955	0.973	0.957	0.965	0.822	0.826
SomaticSeq**	0.983	0.960	0.949	0.846	0.651	0.432	0.552	0.546	0.543	0.515	0.450	0.351	0.707	0.696	0.691	0.640	0.532	0.387
INDEL																		
Indelocator	0.810	0.334	0.046	0.012	0.000	0.000	0.663	0.448	0.101	0.029	0.001	0.000	0.729	0.382	0.064	0.017	0.001	0.000
VarScan	0.963	0.961	0.403	0.399	0.041	0.036	0.245	0.244	0.119	0.118	0.013	0.012	0.390	0.390	0.184	0.182	0.020	0.018
VarScan + Filter	0.417	0.417	0.177	0.176	0.018	0.016	0.245	0.245	0.121	0.121	0.014	0.012	0.309	0.309	0.144	0.143	0.015	0.014
VarDict	0.852	0.631	0.815	0.579	0.612	0.299	0.268	0.213	0.259	0.199	0.208	0.114	0.408	0.319	0.393	0.296	0.311	0.165
Union of Tools	0.979	0.976	0.871	0.764	0.765	0.497	0.084	0.084	0.075	0.067	0.067	0.044	0.155	0.154	0.139	0.134	0.123	0.082
SomaticSeq	0.864	0.864	0.747	0.668	0.688	0.399	0.966	0.961	0.971	0.940	0.917	0.951	0.912	0.910	0.844	0.793	0.786	0.562
SomaticSeq*	0.839	0.858	0.782	0.695	0.596	0.403	0.998	0.998	0.998	0.998	0.998	0.997	0.912	0.923	0.877	0.819	0.746	0.574
SomaticSeq**	0.542	0.416	0.556	0.322	0.445	0.169	0.785	0.737	0.789	0.685	0.750	0.532	0.642	0.531	0.652	0.438	0.559	0.256

TABLE 3. Prior probability of somatic mutation is enforced to be 1 in a million in order to get a more realistic performance. VAF_N and VAF_T stand for variant allele frequencies of the Normal and the Tumor, respectively. VarScan+Filter and Sniper+Filter are subsets of VarScan2 and SomaticSniper output, respectively, with author-recommended false positive filters applied. The trained model for SomaticSeq* is the combined settings of the *in silico* titration randomly split into half, which has 6 times the data size used during cross validation. The trained model for SomaticSeq** is the DREAM Challenge with settings A and B combined, with dbSNP features discarded.

10X	Recall				Precision				F_1 Score			
VAF	5%	10%	20%	40%	5%	10%	20%	40%	5%	10%	20%	40%
MuTect	0.028	0.137	0.457	0.830	0.079	0.297	0.584	0.719	0.041	0.188	0.513	0.770
VarScan	0.031	0.129	0.481	0.876	0.001	0.005	0.019	0.035	0.002	0.010	0.037	0.066
VarScan+Filter	0.009	0.059	0.279	0.619	0.010	0.061	0.233	0.403	0.009	0.060	0.254	0.488
SomaticSniper	0.008	0.079	0.386	0.850	0.003	0.030	0.133	0.252	0.004	0.044	0.198	0.389
Sniper+Filter	0.006	0.051	0.259	0.610	0.010	0.086	0.325	0.531	0.007	0.064	0.288	0.568
JointSNVMix	0.006	0.070	0.395	0.882	0.001	0.017	0.088	0.177	0.002	0.027	0.144	0.294
VarDict	0.022	0.122	0.418	0.737	0.004	0.023	0.074	0.123	0.007	0.038	0.125	0.211
Union of Tools	0.064	0.227	0.613	0.936	0.002	0.007	0.018	0.027	0.004	0.013	0.035	0.053
SomaticSeq	0.014	0.140	0.487	0.857	0.722	0.889	0.952	0.988	0.028	0.242	0.645	0.918

Table 4. SomaticSpike. Tumor sequencing depth = 10X. Prior probability of somatic mutation is enforced to be 1 in a million in order to get a more realistic performance.

20X	Recall				Precision				F_1 Score			
VAF	5%	10%	20%	40%	5%	10%	20%	40%	5%	10%	20%	40%
MuTect	0.141	0.426	0.811	0.959	0.135	0.321	0.473	0.515	0.138	0.366	0.598	0.670
VarScan	0.006	0.080	0.533	0.976	0.001	0.007	0.042	0.074	0.001	0.012	0.078	0.138
VarScan+Filter	0.004	0.065	0.419	0.806	0.002	0.041	0.218	0.349	0.003	0.050	0.287	0.487
SomaticSniper	0.009	0.131	0.628	0.976	0.002	0.034	0.143	0.206	0.004	0.054	0.233	0.340
Sniper+Filter	0.007	0.104	0.492	0.809	0.008	0.099	0.344	0.463	0.008	0.101	0.405	0.589
JointSNVMix	0.016	0.331	0.847	0.983	0.002	0.041	0.098	0.111	0.004	0.072	0.175	0.200
VarDict	0.044	0.224	0.640	0.812	0.006	0.031	0.084	0.104	0.011	0.055	0.149	0.185
Union of Tools	0.174	0.514	0.902	0.992	0.005	0.015	0.026	0.029	0.010	0.029	0.051	0.056
SomaticSeq	0.115	0.395	0.816	0.977	0.860	0.932	0.971	0.996	0.203	0.555	0.887	0.986

Table 5. SomaticSpike. Tumor sequencing depth = 20X. Prior probability of somatic mutation is enforced to be 1 in a million in order to get a more realistic performance.

30X	Recall				Precision				F_1 Score			
VAF	5%	10%	20%	40%	5%	10%	20%	40%	5%	10%	20%	40%
MuTect	0.265	0.649	0.911	0.982	0.152	0.306	0.382	0.400	0.194	0.416	0.538	0.568
VarScan	0.002	0.043	0.539	0.991	0.000	0.005	0.055	0.096	0.000	0.008	0.100	0.176
VarScan+Filter	0.002	0.038	0.442	0.822	0.001	0.022	0.205	0.324	0.001	0.028	0.280	0.465
SomaticSniper	0.010	0.150	0.750	0.992	0.002	0.035	0.153	0.193	0.004	0.056	0.254	0.323
Sniper+Filter	0.007	0.128	0.611	0.823	0.006	0.102	0.351	0.422	0.007	0.113	0.446	0.557
JointSNVMix	0.025	0.580	0.947	0.992	0.002	0.051	0.081	0.084	0.004	0.094	0.149	0.155
VarDict	0.059	0.312	0.735	0.826	0.008	0.040	0.088	0.098	0.014	0.070	0.158	0.175
Union of Tools	0.302	0.727	0.968	0.998	0.008	0.019	0.025	0.025	0.015	0.036	0.048	0.049
SomaticSeq	0.213	0.615	0.911	0.988	0.861	0.946	0.981	0.998	0.342	0.745	0.945	0.993

Table 6. SomaticSpike. Tumor sequencing depth = 30X. Prior probability of somatic mutation is enforced to be 1 in a million in order to get a more realistic performance.

40X	Recall				Precision				F_1 Score			
VAF	5%	10%	20%	40%	5%	10%	20%	40%	5%	10%	20%	40%
MuTect	0.328	0.752	0.953	0.985	0.150	0.288	0.339	0.347	0.206	0.417	0.500	0.513
VarScan	0.001	0.029	0.551	0.995	0.000	0.004	0.064	0.110	0.000	0.006	0.115	0.198
VarScan+Filter	0.001	0.027	0.452	0.837	0.000	0.014	0.198	0.314	0.000	0.019	0.278	0.457
SomaticSniper	0.005	0.158	0.825	0.996	0.001	0.036	0.164	0.191	0.002	0.059	0.273	0.321
Sniper+Filter	0.005	0.136	0.685	0.838	0.004	0.099	0.356	0.404	0.004	0.115	0.469	0.545
JointSNVMix	0.022	0.744	0.975	0.992	0.002	0.053	0.069	0.070	0.003	0.100	0.129	0.131
VarDict	0.050	0.363	0.778	0.850	0.007	0.049	0.100	0.108	0.012	0.087	0.177	0.192
Union of Tools	0.357	0.826	0.989	0.999	0.009	0.021	0.025	0.025	0.018	0.041	0.049	0.049
SomaticSeq	0.268	0.724	0.941	0.990	0.858	0.960	0.985	0.999	0.409	0.826	0.962	0.994

Table 7. SomaticSpike. Tumor sequencing depth = 40X. Prior probability of somatic mutation is enforced to be 1 in a million in order to get a more realistic performance.

50X	Recall				Precision				F_1 Score			
VAF	5%	10%	20%	40%	5%	10%	20%	40%	5%	10%	20%	40%
MuTect	0.388	0.817	0.967	0.989	0.159	0.286	0.321	0.326	0.226	0.423	0.482	0.490
VarScan	0.000	0.015	0.582	0.997	0.000	0.002	0.073	0.119	0.000	0.004	0.130	0.213
VarScan+Filter	0.000	0.012	0.492	0.829	0.000	0.007	0.207	0.306	0.000	0.009	0.292	0.447
SomaticSniper	0.005	0.138	0.869	0.997	0.001	0.032	0.175	0.195	0.002	0.052	0.291	0.326
Sniper+Filter	0.005	0.120	0.736	0.829	0.004	0.086	0.367	0.395	0.004	0.101	0.490	0.535
JointSNVMix	0.020	0.864	0.987	0.993	0.001	0.055	0.063	0.063	0.002	0.104	0.118	0.119
VarDict	0.059	0.380	0.808	0.848	0.009	0.054	0.109	0.114	0.015	0.095	0.192	0.201
Union of Tools	0.418	0.895	0.994	0.998	0.011	0.023	0.026	0.026	0.022	0.046	0.051	0.051
SomaticSeq	0.324	0.708	0.946	0.993	0.873	0.968	0.988	1.000	0.472	0.881	0.966	0.996

Table 8. SomaticSpike. Tumor sequencing depth = 50X. Prior probability of somatic mutation is enforced to be 1 in a million in order to get a more realistic performance.

Consensus	DC3A	DC3B	DC3C	DC3D	N_0T_{50}	$N_{2.5}T_{50}$	N_0T_{25}	$N_{2.5}T_{25}$	N_0T_{15}	$N_{2.5}T_{15}$
MV	0.787	0.648	0.626	0.477	0.698	0.569	0.488	0.364	0.079	0.035
MJ	0.889	0.764	0.760	0.620	0.593	0.476	0.571	0.452	0.428	0.307
MS	0.842	0.709	0.672	0.525	0.656	0.531	0.553	0.427	0.191	0.111
MD	0.899	0.772	0.844	0.699	0.527	0.410	0.514	0.396	0.441	0.292
VJ	0.677	0.637	0.528	0.480	0.344	0.328	0.224	0.205	0.032	0.023
VS	0.665	0.645	0.511	0.482	0.411	0.409	0.271	0.266	0.039	0.035
VD	0.796	0.703	0.634	0.524	0.693	0.565	0.487	0.363	0.078	0.035
JS	0.724	0.691	0.568	0.532	0.390	0.372	0.322	0.301	0.101	0.084
JD	0.908	0.821	0.780	0.677	0.606	0.487	0.591	0.469	0.453	0.315
SD	0.848	0.764	0.679	0.580	0.635	0.513	0.538	0.413	0.185	0.106
MVJ	0.788	0.649	0.625	0.475	0.700	0.571	0.489	0.365	0.079	0.036
MVS	0.782	0.640	0.611	0.460	0.724	0.592	0.508	0.380	0.082	0.037
MVD	0.810	0.667	0.645	0.490	0.813	0.661	0.585	0.431	0.099	0.042
MJS	0.838	0.705	0.669	0.522	0.685	0.557	0.579	0.449	0.203	0.118
MJD	0.925	0.795	0.794	0.647	0.743	0.597	0.717	0.568	0.551	0.380
MSD	0.862	0.725	0.690	0.536	0.746	0.600	0.637	0.486	0.229	0.128
VJS	0.715	0.674	0.553	0.506	0.429	0.409	0.283	0.261	0.041	0.030
VJD	0.800	0.700	0.636	0.520	0.697	0.568	0.490	0.365	0.079	0.035
VSD	0.786	0.692	0.617	0.506	0.714	0.583	0.503	0.375	0.081	0.036
JSD	0.851	0.759	0.682	0.576	0.670	0.544	0.571	0.440	0.199	0.114
MVJS	0.778	0.637	0.608	0.458	0.725	0.593	0.509	0.381	0.082	0.037
MVJD	0.805	0.661	0.639	0.485	0.814	0.662	0.586	0.432	0.099	0.043
MVSD	0.792	0.647	0.621	0.465	0.822	0.670	0.592	0.437	0.100	0.043
MJSD	0.857	0.721	0.686	0.533	0.782	0.633	0.670	0.514	0.245	0.138
VJSD	0.785	0.685	0.616	0.501	0.715	0.584	0.504	0.376	0.081	0.036
MVJSD	0.788	0.644	0.618	0.462	0.823	0.670	0.592	0.438	0.100	0.043
$\geq$ 2 tools	0.672	0.651	0.623	0.584	0.247	0.246	0.244	0.235	0.208	0.157
$\geq$ 3 tools	0.833	0.793	0.708	0.655	0.380	0.362	0.364	0.325	0.260	0.181
$\geq$ 4 tools	0.860	0.765	0.696	0.581	0.610	0.501	0.513	0.399	0.174	0.099
5 tools	0.788	0.644	0.618	0.462	0.823	0.670	0.592	0.438	0.100	0.043
SomaticSeq	0.964	0.958	0.932	0.905	0.955	0.952	0.946	0.942	0.823	0.805

Table 9. F_1 scores of simple consensus. M: MuTect. V: VarScan2. J: JointSNVMix2. S: SomaticSniper. D: VarDict with relaxed filters. MJ represent all calls intersected by MuTect (M) and JointSNVMix2 (J). The first four columns represent the four DREAM Challenge Stage 3 (DC3) settings. The last six columns represent the six *in silico* titration settings. The subscripts denote the expected VAF (%) of the Normal (N) and Tumor (T).

Consensus	DC3A	DC3B	DC3C	DC3D	N_0T_{50}	$N_{2.5}T_{50}$	N_0T_{25}	$N_{2.5}T_{25}$	N_0T_{15}	$N_{2.5}T_{15}$
MV	0.696	0.514	0.489	0.336	0.991	0.734	0.596	0.411	0.076	0.033
MJ	0.883	0.681	0.676	0.496	0.991	0.734	0.938	0.687	0.639	0.425
MS	0.767	0.580	0.534	0.375	0.990	0.734	0.775	0.551	0.215	0.119
MD	0.909	0.700	0.813	0.599	0.983	0.708	0.949	0.677	0.777	0.468
VJ	0.694	0.633	0.486	0.428	1.000	0.943	0.606	0.550	0.078	0.056
VS	0.671	0.642	0.461	0.427	1.000	0.992	0.605	0.593	0.077	0.069
VD	0.696	0.571	0.489	0.374	0.988	0.733	0.600	0.413	0.076	0.033
JS	0.768	0.714	0.536	0.491	1.000	0.942	0.791	0.730	0.219	0.180
JD	0.887	0.741	0.682	0.545	0.988	0.733	0.954	0.697	0.667	0.425
SD	0.769	0.645	0.537	0.427	0.988	0.733	0.783	0.553	0.216	0.119
MVJ	0.685	0.506	0.478	0.328	0.991	0.734	0.596	0.411	0.076	0.033
MVS	0.663	0.487	0.455	0.309	0.990	0.734	0.594	0.410	0.075	0.033
MVD	0.687	0.505	0.481	0.327	0.983	0.708	0.592	0.394	0.075	0.031
MJS	0.759	0.574	0.529	0.372	0.990	0.734	0.775	0.551	0.215	0.119
MJD	0.872	0.669	0.668	0.485	0.983	0.708	0.931	0.660	0.632	0.391
MSD	0.762	0.573	0.530	0.369	0.983	0.708	0.771	0.530	0.214	0.113
VJS	0.665	0.608	0.458	0.405	1.000	0.942	0.605	0.549	0.077	0.056
VJD	0.686	0.555	0.481	0.362	0.988	0.733	0.599	0.412	0.076	0.033
VSD	0.665	0.543	0.458	0.348	0.988	0.733	0.598	0.411	0.075	0.032
JSD	0.761	0.630	0.532	0.416	0.988	0.733	0.783	0.553	0.216	0.119
MVJS	0.658	0.483	0.451	0.306	0.990	0.734	0.594	0.410	0.075	0.033
MVJD	0.678	0.498	0.474	0.322	0.983	0.708	0.592	0.394	0.075	0.031
MVSD	0.659	0.481	0.453	0.304	0.983	0.708	0.591	0.394	0.074	0.031
MJSD	0.755	0.568	0.526	0.366	0.983	0.708	0.771	0.530	0.214	0.113
VJSD	0.659	0.532	0.454	0.341	0.988	0.733	0.598	0.411	0.075	0.032
MVJSD	0.654	0.477	0.449	0.302	0.983	0.708	0.591	0.394	0.074	0.031
$\geq$ 2 tools	0.940	0.898	0.841	0.766	1.000	0.992	0.984	0.941	0.821	0.606
$\geq$ 3 tools	0.901	0.830	0.691	0.615	1.000	0.943	0.950	0.826	0.637	0.423
$\geq$ 4 tools	0.795	0.652	0.561	0.431	0.995	0.758	0.783	0.564	0.216	0.118
5 tools	0.654	0.477	0.449	0.302	0.983	0.708	0.591	0.394	0.074	0.031
SomaticSeq	0.937	0.923	0.875	0.832	0.947	0.936	0.917	0.903	0.721	0.684

TABLE 10. Sensitivity of a simple consensus approach. M: MuTect. V: VarScan2. J: JointSNVMix2. S: SomaticSniper. D: VarDict with relaxed filters. MJ represent all calls intersected by MuTect (M) and JointSNVMix2 (J). The first four columns represent the four DREAM Challenge Stage 3 (DC3) settings. The last six columns represent the six *in silico* titration settings. The subscripts denote the expected VAF (%) of the Normal (N) and Tumor (T).

Consensus	DC3A	DC3B	DC3C	DC3D	N_0T_{50}	$N_{2.5}T_{50}$	N_0T_{25}	$N_{2.5}T_{25}$	N_0T_{15}	$N_{2.5}T_{15}$
MV	0.905	0.875	0.869	0.821	0.539	0.464	0.413	0.327	0.082	0.038
MJ	0.896	0.869	0.868	0.828	0.423	0.353	0.410	0.337	0.322	0.240
MS	0.933	0.914	0.907	0.873	0.491	0.416	0.430	0.349	0.173	0.104
MD	0.889	0.860	0.877	0.840	0.360	0.289	0.352	0.280	0.308	0.212
VJ	0.661	0.640	0.578	0.546	0.208	0.198	0.137	0.126	0.020	0.015
VS	0.659	0.649	0.571	0.552	0.259	0.257	0.174	0.172	0.026	0.023
VD	0.929	0.914	0.902	0.875	0.534	0.459	0.410	0.324	0.081	0.037
JS	0.685	0.669	0.603	0.582	0.242	0.232	0.202	0.189	0.065	0.055
JD	0.931	0.919	0.912	0.893	0.437	0.365	0.428	0.353	0.343	0.250
SD	0.946	0.936	0.924	0.906	0.468	0.394	0.410	0.330	0.161	0.095
MVJ	0.928	0.905	0.900	0.861	0.541	0.467	0.415	0.329	0.083	0.038
MVS	0.952	0.936	0.931	0.902	0.571	0.496	0.444	0.355	0.091	0.042
MVD	0.987	0.982	0.981	0.973	0.693	0.620	0.577	0.476	0.147	0.067
MJS	0.935	0.916	0.910	0.876	0.523	0.449	0.462	0.379	0.192	0.117
MJD	0.983	0.979	0.979	0.971	0.597	0.516	0.584	0.498	0.488	0.371
MSD	0.990	0.987	0.986	0.980	0.601	0.521	0.542	0.448	0.247	0.148
VJS	0.772	0.756	0.700	0.674	0.273	0.261	0.185	0.171	0.028	0.021
VJD	0.958	0.948	0.941	0.923	0.539	0.464	0.415	0.327	0.082	0.037
VSD	0.962	0.954	0.946	0.930	0.558	0.484	0.433	0.345	0.087	0.040
JSD	0.964	0.957	0.949	0.936	0.507	0.433	0.449	0.366	0.184	0.110
MVJS	0.953	0.937	0.933	0.904	0.572	0.497	0.445	0.356	0.092	0.042
MVJD	0.989	0.984	0.984	0.976	0.695	0.622	0.579	0.478	0.148	0.067
MVSD	0.992	0.989	0.989	0.983	0.707	0.635	0.592	0.492	0.154	0.070
MJSD	0.991	0.988	0.987	0.981	0.650	0.572	0.593	0.500	0.287	0.176
VJSD	0.970	0.963	0.957	0.944	0.560	0.486	0.435	0.347	0.088	0.040
MVJSD	0.992	0.990	0.989	0.984	0.708	0.636	0.593	0.493	0.154	0.070
$\geq$ 2 tools	0.522	0.511	0.495	0.471	0.141	0.140	0.139	0.134	0.119	0.091
$\geq$ 3 tools	0.774	0.759	0.725	0.701	0.235	0.224	0.226	0.202	0.163	0.115
$\geq$ 4 tools	0.938	0.925	0.914	0.891	0.440	0.375	0.382	0.308	0.146	0.085
5 tools	0.992	0.990	0.989	0.984	0.708	0.636	0.593	0.493	0.154	0.070
SomaticSeq	0.992	0.997	0.996	0.994	0.963	0.969	0.978	0.985	0.959	0.978

TABLE 11. Precision of simple consensus approach. M: MuTect. V: VarScan2. J: JointSNVMix2. S: SomaticSniper. D: VarDict with relaxed filters. MJ represent all calls intersected by MuTect (M) and JointSNVMix2 (J). The first four columns represent the four DREAM Challenge Stage 3 (DC3) settings. The last six columns represent the six *in silico* titration settings. The subscripts denote the expected VAF (%) of the Normal (N) and Tumor (T).

	CLL1		COLO829	
Consensus	Recall	# Calls	Recall	# Calls
MV	0.865	2,323	0.985	37,106
MJ	0.883	3,363	0.993	39,898
MS	0.888	2,503	0.993	37,495
MD	0.877	3,669	0.866	38,059
VJ	0.866	5,386	0.987	43,963
VS	0.880	6,872	0.987	45,072
VD	0.854	2,731	0.859	35,900
JS	0.889	7,061	0.996	44,313
JD	0.872	3,444	0.868	38,034
SD	0.874	2,821	0.868	36,102
MVJ	0.855	2,149	0.985	36,851
MVS	0.864	2,098	0.985	36,619
MVD	0.849	1,845	0.857	34,327
MJS	0.877	2,416	0.993	37,376
MJD	0.868	2,230	0.866	35,751
MSD	0.871	1,952	0.866	34,740
VJS	0.865	4,470	0.987	41,038
VJD	0.846	2,271	0.859	35,331
VSD	0.851	2,327	0.859	35,180
JSD	0.866	2,483	0.868	35,799
MVJS	0.855	2,042	0.985	36,537
MVJD	0.842	1,782	0.857	34,223
MVSD	0.848	1,779	0.857	34,139
MJSD	0.863	1,913	0.866	34,673
VJSD	0.845	2,117	0.859	34,988
MVJSD	0.842	1,749	0.857	34,086
$\geq$ 2 tools	0.916	13,594	0.996	57,254
$\geq$ 3 tools	0.904	5,836	0.996	43,848
$\geq$ 4 tools	0.886	2,637	0.996	38,216
5 tools	0.842	1,749	0.857	34,086
SomaticSeq	0.892	2,320	0.996	37,452

Table 12. Sensitivity and call set size of prediction of CLL1 (chronic lymphocytic leukemia) and COLO829 (melanoma) based on a simple consensus.

SNV	DC3A	DC3B	DC3C	DC3D	N_0T_{50}	$N_{2.5}T_{50}$	N_0T_{25}	$N_{2.5}T_{25}$	N_0T_{15}	$N_{2.5}T_{15}$
M	0.951	0.827	0.899	0.760	0.928	0.790	0.887	0.738	0.722	0.569
V	0.811	0.792	0.649	0.612	0.922	0.938	0.699	0.693	0.096	0.098
J	0.937	0.895	0.801	0.760	0.932	0.915	0.907	0.882	0.729	0.700
S	0.867	0.850	0.694	0.680	0.931	0.940	0.836	0.848	0.319	0.325
D	0.954	0.886	0.918	0.809	0.941	0.794	0.925	0.769	0.814	0.583
MV	0.950	0.921	0.900	0.840	0.939	0.945	0.905	0.866	0.716	0.612
MJ	0.958	0.920	0.903	0.851	0.931	0.914	0.906	0.879	0.768	0.747
MS	0.952	0.928	0.898	0.860	0.941	0.941	0.903	0.902	0.724	0.662
MD	0.964	0.890	0.933	0.843	0.948	0.813	0.927	0.793	0.807	0.649
VJ	0.935	0.915	0.804	0.777	0.937	0.936	0.906	0.902	0.745	0.718
VS	0.880	0.865	0.713	0.701	0.941	0.944	0.844	0.845	0.335	0.328
VD	0.953	0.943	0.921	0.869	0.950	0.946	0.927	0.900	0.813	0.615
JS	0.935	0.920	0.805	0.784	0.935	0.944	0.919	0.917	0.753	0.732
JD	0.956	0.939	0.916	0.870	0.942	0.917	0.943	0.913	0.819	0.759
SD	0.956	0.947	0.915	0.875	0.947	0.947	0.939	0.928	0.804	0.679
MVJ	0.956	0.931	0.900	0.864	0.945	0.947	0.926	0.915	0.779	0.760
MVS	0.951	0.931	0.900	0.858	0.950	0.950	0.901	0.902	0.721	0.658
MVD	0.962	0.950	0.932	0.890	0.952	0.955	0.929	0.901	0.809	0.686
MJS	0.958	0.936	0.903	0.867	0.937	0.944	0.928	0.915	0.778	0.775
MJD	0.960	0.941	0.931	0.891	0.951	0.931	0.940	0.916	0.829	0.783
MSD	0.963	0.947	0.931	0.898	0.951	0.955	0.934	0.923	0.808	0.716
VJS	0.939	0.923	0.805	0.789	0.933	0.949	0.927	0.927	0.757	0.738
VJD	0.956	0.951	0.923	0.884	0.951	0.952	0.942	0.937	0.822	0.769
VSD	0.955	0.950	0.918	0.875	0.950	0.955	0.937	0.925	0.818	0.668
JSD	0.957	0.951	0.921	0.885	0.945	0.948	0.938	0.950	0.819	0.780
MVJS	0.958	0.938	0.899	0.869	0.943	0.947	0.929	0.932	0.781	0.781
MVJD	0.963	0.955	0.931	0.901	0.948	0.958	0.937	0.934	0.824	0.793
MVSD	0.964	0.958	0.931	0.899	0.949	0.955	0.929	0.932	0.810	0.724
MJSD	0.962	0.952	0.927	0.902	0.950	0.952	0.942	0.947	0.825	0.807
VJSD	0.956	0.957	0.920	0.885	0.947	0.957	0.946	0.944	0.826	0.778
MVJSD	0.964	0.958	0.932	0.905	0.955	0.952	0.946	0.942	0.823	0.805
INDEL										
M	0.266	0.135	0.048	0.013	0.831	0.393	0.051	0.002	0.000	0.000
V	0.489	0.472	0.279	0.268	0.906	0.897	0.517	0.520	0.052	0.045
D	0.877	0.833	0.852	0.762	0.834	0.697	0.830	0.681	0.737	0.532
MV	0.506	0.487	0.284	0.264	0.908	0.901	0.509	0.519	0.739	0.046
MD	0.878	0.834	0.852	0.760	0.857	0.711	0.834	0.680	0.748	0.533
VD	0.875	0.869	0.848	0.786	0.910	0.891	0.850	0.793	0.738	0.556
MVD	0.874	0.868	0.855	0.788	0.912	0.910	0.844	0.793	0.786	0.562

Table 13. F1 scores of every possible combination of individual callers, followed by the same machine learning algorithm. M: MuTect/Indelocator. V: VarScan2. J: JointSNVMix2. S: SomaticSniper. D: VarDict with relaxed filters. MJ represent all calls intersected by MuTect (M) and JointSNVMix2 (J). The first four columns represent the four DREAM Challenge Stage 3 (DC3) settings. The last six columns represent the six *in silico* titration settings. The subscripts denote the expected VAF (%) of the Normal (N) and Tumor (T).

SNV	DC3A	DC3B	DC3C	DC3D	N_0T_{50}	$N_{2.5}T_{50}$	N_0T_{25}	$N_{2.5}T_{25}$	N_0T_{15}	$N_{2.5}T_{15}$
M	0.917	0.714	0.835	0.626	0.913	0.676	0.834	0.605	0.606	0.412
V	0.689	0.664	0.484	0.445	0.911	0.927	0.558	0.544	0.051	0.052
J	0.889	0.818	0.674	0.620	0.911	0.873	0.870	0.820	0.594	0.551
S	0.770	0.744	0.534	0.517	0.911	0.920	0.737	0.749	0.191	0.195
D	0.918	0.795	0.849	0.680	0.928	0.681	0.889	0.636	0.717	0.420
MV	0.918	0.869	0.834	0.742	0.918	0.930	0.858	0.785	0.598	0.457
MJ	0.930	0.862	0.839	0.758	0.914	0.873	0.869	0.813	0.652	0.616
MS	0.922	0.877	0.832	0.772	0.925	0.921	0.848	0.840	0.603	0.513
MD	0.938	0.804	0.877	0.730	0.935	0.704	0.893	0.668	0.710	0.490
VJ	0.887	0.857	0.679	0.644	0.921	0.917	0.872	0.857	0.609	0.574
VS	0.792	0.772	0.559	0.543	0.933	0.926	0.744	0.743	0.202	0.197
VD	0.916	0.894	0.854	0.770	0.941	0.929	0.888	0.830	0.715	0.452
JS	0.887	0.862	0.680	0.652	0.925	0.927	0.879	0.869	0.618	0.589
JD	0.922	0.886	0.846	0.772	0.926	0.872	0.913	0.854	0.716	0.621
SD	0.923	0.901	0.843	0.780	0.937	0.926	0.904	0.877	0.703	0.524
MVJ	0.926	0.884	0.834	0.778	0.933	0.932	0.893	0.871	0.665	0.629
MVS	0.919	0.885	0.835	0.769	0.942	0.934	0.843	0.840	0.605	0.510
MVD	0.934	0.908	0.876	0.805	0.943	0.941	0.893	0.833	0.712	0.534
MJS	0.930	0.889	0.842	0.783	0.921	0.922	0.892	0.862	0.661	0.649
MJD	0.930	0.890	0.875	0.807	0.940	0.895	0.909	0.855	0.732	0.651
MSD	0.935	0.901	0.874	0.819	0.939	0.938	0.896	0.872	0.709	0.571
VJS	0.895	0.870	0.680	0.659	0.918	0.936	0.891	0.884	0.622	0.594
VJD	0.922	0.909	0.858	0.794	0.942	0.939	0.911	0.895	0.720	0.633
VSD	0.920	0.908	0.849	0.779	0.938	0.943	0.903	0.875	0.726	0.513
JSD	0.924	0.909	0.854	0.795	0.930	0.928	0.899	0.917	0.717	0.648
MVJS	0.931	0.896	0.831	0.787	0.930	0.931	0.890	0.891	0.666	0.655
MVJD	0.935	0.916	0.874	0.824	0.932	0.945	0.903	0.891	0.725	0.667
MVSD	0.938	0.922	0.874	0.820	0.938	0.939	0.886	0.884	0.712	0.580
MJSD	0.934	0.910	0.868	0.825	0.940	0.932	0.906	0.915	0.725	0.685
VJSD	0.922	0.920	0.852	0.796	0.939	0.946	0.914	0.904	0.727	0.644
MVJSD	0.937	0.923	0.875	0.832	0.947	0.936	0.917	0.903	0.721	0.684
INDEL										
M	0.156	0.073	0.025	0.007	0.766	0.261	0.026	0.001	0.000	0.000
V	0.334	0.318	0.165	0.157	0.861	0.845	0.354	0.356	0.027	0.023
D	0.814	0.722	0.750	0.622	0.775	0.563	0.735	0.529	0.610	0.370
MV	0.350	0.330	0.168	0.154	0.861	0.850	0.348	0.356	0.610	0.024
MD	0.815	0.723	0.751	0.619	0.788	0.570	0.741	0.528	0.619	0.372
VD	0.809	0.777	0.744	0.655	0.860	0.837	0.756	0.668	0.609	0.393
MVD	0.807	0.776	0.754	0.656	0.864	0.864	0.747	0.668	0.688	0.399

TABLE 14. Recalls of every possible combination of individual callers, followed by the same machine learning algorithm. M: MuTect/Indelocator. V: VarScan2. J: JointSNVMix2. S: SomaticSniper. D: VarDict with relaxed filters. MJ represent all calls intersected by MuTect (M) and JointSNVMix2 (J). The first four columns represent the four DREAM Challenge Stage 3 (DC3) settings. The last six columns represent the six *in silico* titration settings. The subscripts denote the expected VAF (%) of the Normal (N) and Tumor (T).

SNV	DC3A	DC3B	DC3C	DC3D	N_0T_{50}	$N_{2.5}T_{50}$	N_0T_{25}	$N_{2.5}T_{25}$	N_0T_{15}	$N_{2.5}T_{15}$
M	0.987	0.983	0.974	0.966	0.943	0.949	0.948	0.946	0.894	0.919
V	0.986	0.982	0.985	0.982	0.932	0.950	0.937	0.954	0.873	0.911
J	0.990	0.988	0.985	0.982	0.953	0.962	0.948	0.955	0.942	0.961
S	0.992	0.991	0.992	0.990	0.951	0.960	0.967	0.978	0.975	0.978
D	0.993	0.999	1.000	0.999	0.953	0.952	0.964	0.972	0.941	0.954
MV	0.986	0.980	0.977	0.968	0.961	0.961	0.958	0.966	0.894	0.929
MJ	0.987	0.985	0.977	0.971	0.949	0.959	0.947	0.957	0.935	0.951
MS	0.985	0.986	0.975	0.970	0.958	0.963	0.966	0.973	0.906	0.934
MD	0.992	0.998	0.995	0.997	0.961	0.962	0.965	0.976	0.936	0.960
VJ	0.988	0.982	0.984	0.980	0.953	0.957	0.943	0.952	0.958	0.960
VS	0.989	0.985	0.987	0.986	0.950	0.963	0.975	0.980	0.982	0.985
VD	0.993	0.998	0.999	0.998	0.959	0.963	0.969	0.983	0.941	0.964
JS	0.988	0.987	0.986	0.984	0.946	0.962	0.962	0.971	0.962	0.969
JD	0.992	0.998	0.999	0.997	0.959	0.966	0.974	0.981	0.957	0.978
SD	0.992	0.998	1.000	0.998	0.957	0.970	0.977	0.986	0.940	0.965
MVJ	0.987	0.983	0.976	0.971	0.957	0.963	0.962	0.965	0.941	0.961
MVS	0.986	0.982	0.976	0.971	0.957	0.967	0.967	0.974	0.892	0.930
MVD	0.991	0.996	0.995	0.996	0.962	0.970	0.969	0.981	0.937	0.960
MJS	0.988	0.987	0.975	0.970	0.953	0.967	0.968	0.976	0.945	0.963
MJD	0.993	0.997	0.996	0.994	0.962	0.970	0.973	0.987	0.956	0.981
MSD	0.992	0.997	0.995	0.994	0.963	0.973	0.977	0.982	0.939	0.961
VJS	0.987	0.984	0.984	0.983	0.947	0.962	0.966	0.976	0.968	0.974
VJD	0.992	0.997	0.999	0.996	0.961	0.967	0.976	0.983	0.959	0.979
VSD	0.992	0.997	0.999	0.997	0.961	0.968	0.974	0.982	0.939	0.960
JSD	0.993	0.997	0.999	0.996	0.960	0.970	0.981	0.985	0.956	0.980
MVJS	0.987	0.985	0.978	0.970	0.956	0.964	0.973	0.977	0.944	0.967
MVJD	0.993	0.996	0.996	0.995	0.964	0.973	0.975	0.981	0.955	0.978
MVSD	0.992	0.996	0.995	0.995	0.962	0.971	0.976	0.985	0.938	0.962
MJSD	0.992	0.997	0.995	0.994	0.960	0.973	0.982	0.982	0.957	0.982
VJSD	0.993	0.997	0.999	0.997	0.956	0.970	0.981	0.987	0.956	0.984
MVJSD	0.992	0.997	0.996	0.994	0.963	0.969	0.978	0.985	0.959	0.978
INDEL										
M	0.906	0.857	0.834	0.674	0.908	0.801	0.838	0.964	-	-
V	0.917	0.913	0.903	0.912	0.955	0.957	0.957	0.955	0.930	0.924
D	0.951	0.984	0.986	0.984	0.904	0.914	0.955	0.957	0.933	0.950
MV	0.915	0.922	0.902	0.906	0.960	0.959	0.949	0.956	0.939	0.937
MD	0.953	0.986	0.986	0.986	0.941	0.944	0.955	0.974	0.944	0.939
VD	0.954	0.985	0.985	0.983	0.967	0.951	0.971	0.977	0.936	0.950
MVD	0.952	0.985	0.986	0.985	0.966	0.961	0.971	0.940	0.917	0.951

TABLE 15. Precisions of every possible combination of individual callers, followed by the same machine learning algorithm. M: MuTect/Indelocator. V: VarScan2. J: JointSNVMix2. S: SomaticSniper. D: VarDict with relaxed filters. MJ represent all calls intersected by MuTect (M) and JointSNVMix2 (J). The first four columns represent the four DREAM Challenge Stage 3 (DC3) settings. The last six columns represent the six *in silico* titration settings. The subscripts denote the expected VAF (%) of the Normal (N) and Tumor (T).

SNV	DC3A	DC3B	DC3C	DC3D	N_0T_{50}	$N_{2.5}T_{50}$	N_0T_{25}	$N_{2.5}T_{25}$	N_0T_{15}	$N_{2.5}T_{15}$
M	0.99800	0.99795	0.99633	0.99631	0.99643	0.99762	0.99701	0.99776	0.99533	0.99763
V	0.99840	0.99800	0.99880	0.99861	0.99569	0.99680	0.99757	0.99829	0.99952	0.99967
J	0.99852	0.99840	0.99834	0.99814	0.99709	0.99775	0.99689	0.99747	0.99760	0.99853
S	0.99897	0.99886	0.99928	0.99917	0.99695	0.99751	0.99836	0.99892	0.99968	0.99971
D	0.99891	0.99991	0.99994	0.99994	0.99704	0.99777	0.99783	0.99882	0.99705	0.99869
MV	0.99780	0.99703	0.99673	0.99588	0.99758	0.99756	0.99758	0.99820	0.99540	0.99771
MJ	0.99801	0.99781	0.99675	0.99632	0.99678	0.99755	0.99685	0.99763	0.99705	0.99795
MS	0.99772	0.99791	0.99641	0.99609	0.99735	0.99767	0.99805	0.99849	0.99594	0.99764
MD	0.99882	0.99967	0.99932	0.99960	0.99753	0.99820	0.99789	0.99894	0.99684	0.99867
VJ	0.99815	0.99744	0.99814	0.99778	0.99707	0.99734	0.99658	0.99718	0.99824	0.99844
VS	0.99849	0.99805	0.99882	0.99876	0.99677	0.99766	0.99875	0.99901	0.99975	0.99980
VD	0.99891	0.99963	0.99985	0.99971	0.99735	0.99769	0.99813	0.99905	0.99707	0.99888
JS	0.99827	0.99815	0.99837	0.99828	0.99657	0.99760	0.99771	0.99830	0.99842	0.99878
JD	0.99883	0.99965	0.99989	0.99962	0.99744	0.99802	0.99844	0.99892	0.99790	0.99909
SD	0.99876	0.99975	0.99993	0.99974	0.99724	0.99811	0.99862	0.99917	0.99707	0.99877
MVJ	0.99800	0.99745	0.99665	0.99609	0.99726	0.99768	0.99767	0.99795	0.99727	0.99832
MVS	0.99777	0.99735	0.99655	0.99618	0.99726	0.99790	0.99810	0.99855	0.99526	0.99749
MVD	0.99866	0.99946	0.99933	0.99943	0.99755	0.99808	0.99812	0.99892	0.99691	0.99855
MJS	0.99811	0.99805	0.99637	0.99598	0.99707	0.99796	0.99808	0.99863	0.99751	0.99836
MJD	0.99889	0.99957	0.99934	0.99922	0.99757	0.99818	0.99835	0.99924	0.99778	0.99920
MSD	0.99877	0.99951	0.99929	0.99924	0.99763	0.99833	0.99861	0.99895	0.99699	0.99850
VJS	0.99803	0.99758	0.99822	0.99811	0.99667	0.99762	0.99793	0.99855	0.99864	0.99896
VJD	0.99878	0.99950	0.99989	0.99952	0.99750	0.99790	0.99852	0.99901	0.99798	0.99912
VSD	0.99876	0.99957	0.99987	0.99956	0.99755	0.99794	0.99840	0.99894	0.99691	0.99861
JSD	0.99893	0.99952	0.99987	0.99952	0.99746	0.99812	0.99884	0.99911	0.99786	0.99913
MVJS	0.99798	0.99770	0.99687	0.99594	0.99721	0.99774	0.99841	0.99864	0.99744	0.99857
MVJD	0.99883	0.99943	0.99946	0.99930	0.99775	0.99828	0.99846	0.99890	0.99777	0.99902
MVSD	0.99875	0.99945	0.99930	0.99926	0.99757	0.99817	0.99861	0.99912	0.99694	0.99852
MJSD	0.99882	0.99951	0.99934	0.99915	0.99747	0.99834	0.99889	0.99893	0.99790	0.99919
VJSD	0.99887	0.99952	0.99987	0.99956	0.99721	0.99808	0.99887	0.99921	0.99783	0.99930
MVJSD	0.99882	0.99950	0.99939	0.99916	0.99761	0.99804	0.99868	0.99911	0.99797	0.99898
SSeq-M	0.06551	0.06619	0.06608	0.06585	0.15993	0.16036	0.16101	0.16144	0.16029	0.16131
SSeq-V	0.09762	0.09829	0.09818	0.09795	0.09185	0.09228	0.09292	0.09335	0.09221	0.09322
SSeq-J	0.17924	0.17991	0.17981	0.17957	0.35654	0.35697	0.35762	0.35804	0.35690	0.35792
SSeq-S	0.02952	0.03019	0.03009	0.02985	0.06162	0.06205	0.06270	0.06313	0.06198	0.06300
SSeq-D	0.09968	0.10035	0.10025	0.10001	0.10473	0.10516	0.10580	0.10623	0.10509	0.10610
INDEL										
M	0.99490	0.99613	0.99844	0.99899	0.99275	0.99397	0.99953	1.00000	0.99891	0.99891
V	0.99055	0.99052	0.99438	0.99521	0.99622	0.99648	0.99851	0.99844	0.99567	0.99982
D	0.98688	0.99636	0.99656	0.99681	0.99238	0.99509	0.99678	0.99779	0.99982	0.99819
MV	0.98984	0.99126	0.99421	0.99495	0.99661	0.99662	0.99826	0.99848	0.99627	0.99985
MD	0.98745	0.99666	0.99671	0.99715	0.99539	0.99683	0.99675	0.99870	0.99656	0.99775
VD	0.98766	0.99626	0.99635	0.99648	0.99729	0.99602	0.99786	0.99852	0.99614	0.99805
MVD	0.98738	0.99634	0.99668	0.99694	0.99718	0.99672	0.99790	0.99603	0.99417	0.99806
SSeq-M	0.12844	0.13741	0.13775	0.13801	0.03430	0.03384	0.03502	0.03315	0.03129	0.03518
SSeq-V	0.10093	0.10990	0.11024	0.11050	0.10439	0.10393	0.10511	0.10324	0.10138	0.10527
SSeq-D	0.09409	0.10305	0.10340	0.10365	0.17622	0.17576	0.17694	0.17507	0.17321	0.17710

Table 16. Negative predictive values (NPV) of all combinations of individual callers, followed by SomaticSeq workflow. M: MuTect/Indelocator. V: VarScan2. J: JointSNVMix2. S: SomaticSniper. D: VarDict with relaxed filters. SSeq-M/V/J/S/D are the differences (improvement) in NPV between 5-tool SomaticSeq and individual callers without SomaticSeq. The first four columns represent the four DREAM Challenge Stage 3 (DC3) settings. The last six columns represent the six *in silico* titration settings.

SNV	DC3A			DC3B			DC3C			DC3D		
Features	5	10	20	5	10	20	5	10	20	5	10	20
Recall	0.843	0.915	0.926	0.798	0.855	0.923	0.727	0.830	0.865	0.663	0.740	0.817
Precision	0.933	0.977	0.989	0.929	0.969	0.990	0.936	0.976	0.991	0.909	0.961	0.988
F1 score	0.886	0.945	0.956	0.858	0.908	0.955	0.818	0.897	0.924	0.767	0.839	0.894
INDEL												
Recall	0.674	0.773	0.810	0.593	0.705	0.767	0.589	0.676	0.752	0.440	0.563	0.642
Precision	0.845	0.941	0.951	0.817	0.930	0.985	0.822	0.936	0.987	0.805	0.923	0.984
F1 score	0.750	0.849	0.875	0.687	0.802	0.862	0.686	0.785	0.853	0.569	0.699	0.777

TABLE 17. Reduced feature set in DREAM Challenge (DC3) cross validations. For easy comparison purposes, the SNV F_1 scores for the full feature set are 0.964, 0.958, 0.932, and 0.905 for DC3A, DC3B, DC3C, and DC3D, respectively. For INDEL F_1 scores, they are 0.874, 0.868, 0.855, and 0.788. (Table 13).

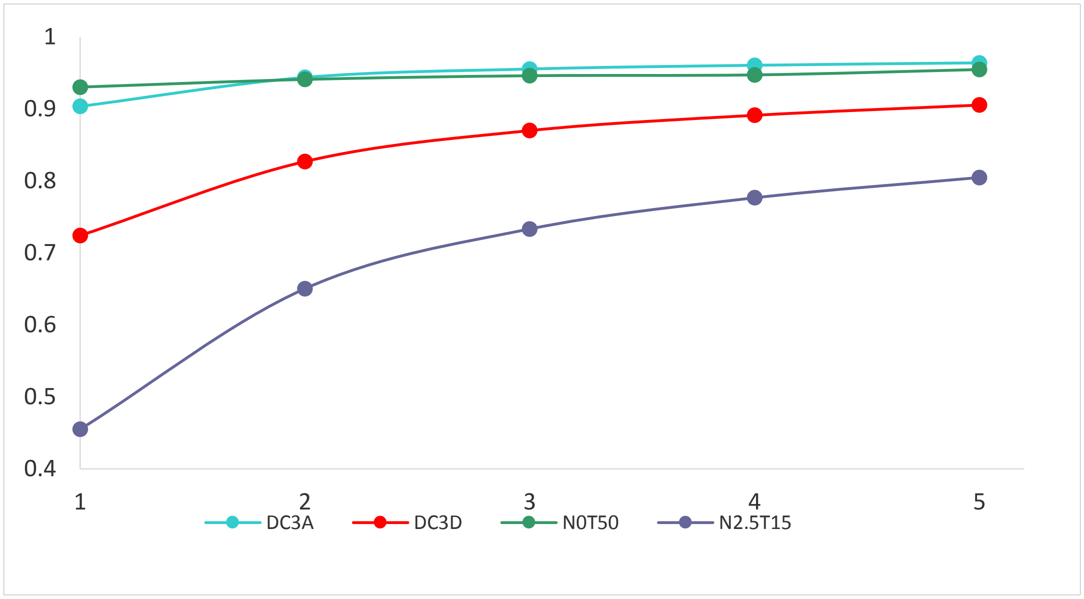

FIGURE 1. The average F_1 scores vs. the number of tools. The gain in accuracy with each addition is the greatest when the data are the mostchallenging (i.e., DC3D and $N_{2.5}T_{15}$), and the least when the data are the simplest (i.e., DC3A and N_0T_{50}). There is also a diminishing return as you add more and more tools.

SNV	DC3A	DC3B	DC3C	DC3D	N_0T_{50}	$N_{2.5}T_{50}$	N_0T_{25}	$N_{2.5}T_{25}$	N_0T_{15}	$N_{2.5}T_{15}$
True Positives	F_1 Score									
0	0.241	0.237	0.226	0.216	0.116	0.116	0.115	0.114	0.106	0.097
10	0.908	0.858	0.863	0.748	0.845	0.823	0.753	0.712	0.571	0.487
20	0.926	0.875	0.878	0.814	0.889	0.873	0.844	0.762	0.601	0.604
50	0.940	0.913	0.897	0.856	0.920	0.910	0.895	0.860	0.678	0.669
100	0.941	0.925	0.912	0.869	0.930	0.919	0.909	0.892	0.747	0.722
200	0.948	0.939	0.919	0.884	0.938	0.933	0.922	0.914	0.774	0.749
500	0.956	0.947	0.925	0.893	0.950	0.943	0.936	0.918	0.808	0.772
1000	0.959	0.952	0.928	0.898	0.952	0.952	0.948	0.940	0.830	0.795
cross validate	0.964	0.958	0.932	0.905	0.955	0.952	0.946	0.942	0.823	0.805
MuTect	0.789	0.671	0.745	0.614	0.400	0.312	0.389	0.302	0.331	0.244
VarScan*	0.573	0.562	0.434	0.413	0.514	0.514	0.345	0.342	0.053	0.048
SomaticSniper*	0.770	0.758	0.607	0.594	0.607	0.604	0.512	0.508	0.177	0.173
JointSNVMix	0.558	0.530	0.459	0.431	0.189	0.179	0.184	0.172	0.133	0.121
VarDict	0.690	0.605	0.607	0.498	0.448	0.359	0.442	0.341	0.363	0.296
	Recall									
0	0.961	0.942	0.892	0.851	1.000	1.000	0.995	0.985	0.917	0.830
10	0.865	0.778	0.807	0.628	0.818	0.805	0.650	0.603	0.442	0.359
20	0.892	0.791	0.798	0.703	0.907	0.849	0.784	0.659	0.465	0.475
50	0.899	0.849	0.820	0.761	0.913	0.910	0.841	0.794	0.542	0.544
100	0.900	0.867	0.845	0.778	0.929	0.925	0.860	0.836	0.618	0.632
200	0.908	0.891	0.853	0.800	0.933	0.934	0.882	0.875	0.654	0.678
500	0.922	0.904	0.864	0.812	0.944	0.943	0.899	0.915	0.699	0.692
1000	0.927	0.912	0.868	0.821	0.944	0.936	0.920	0.898	0.733	0.703
cross validate	0.937	0.923	0.875	0.832	0.947	0.936	0.917	0.903	0.721	0.684
871	Precision									
0	0.138	0.135	0.129	0.124	0.061	0.061	0.061	0.060	0.056	0.051
10	0.955	0.956	0.926	0.925	0.874	0.842	0.893	0.867	0.807	0.756
20	0.962	0.978	0.977	0.968	0.871	0.899	0.913	0.902	0.851	0.829
50	0.985	0.989	0.991	0.979	0.926	0.910	0.956	0.937	0.905	0.867
100	0.986	0.992	0.991	0.984	0.932	0.912	0.963	0.957	0.942	0.840
200	0.992	0.993	0.995	0.988	0.944	0.932	0.967	0.957	0.949	0.837
500	0.993	0.994	0.996	0.991	0.956	0.943	0.977	0.922	0.957	0.873
1000	0.992	0.995	0.996	0.991	0.961	0.969	0.977	0.986	0.956	0.913
cross validate	0.992	0.997	0.996	0.994	0.963	0.969	0.978	0.985	0.959	0.978
T.P. fraction	0.138	0.135	0.129	0.124	0.061	0.061	0.061	0.060	0.056	0.051

Table 18. Reduced size of training data set for SomaticSeq SNVs. Size is defined by the number of true positives. The final row (T.P. fraction) is the fraction of true positives in the training data for each data set, i.e., for DC3A (DREAM Challenge Stage 3, Setting A), the fraction of true positives is 0.138. Thus, when there are 10 true positives, the total training data consisted of $10/0.138 = 73$ calls (of which 10 are true positives and 63 are false positives). 0 means no SomaticSeq training. F_1 score of the individual tools are included for easy comparison. * Author-recommended false positive filters are applied to SomaticSniper and VarScan outputs.

INDEL	DC3A	DC3B	DC3C	DC3D	N_0T_{50}	$N_{2.5}T_{50}$	N_0T_{25}	$N_{2.5}T_{25}$	N_0T_{15}	$N_{2.5}T_{15}$
True Positives	F_1 Score									
0	0.339	0.320	0.310	0.277	0.155	0.154	0.139	0.134	0.123	0.082
10	0.774	0.741	0.748	0.672	0.712	0.677	0.645	0.582	0.483	0.263
20	0.819	0.781	0.786	0.698	0.777	0.748	0.713	0.594	0.553	0.419
50	0.846	0.811	0.820	0.731	0.831	0.810	0.778	0.688	0.556	0.425
100	0.860	0.835	0.831	0.750	0.849	0.836	0.794	0.727	0.660	0.480
200	0.868	0.846	0.838	0.764	0.863	0.859	0.810	0.755	0.707	0.512
500	0.873	0.857	0.844	0.775	0.885	0.887	0.834	0.777	0.732	0.554
1000	0.877	0.863	0.848	0.781	0.911	0.906	0.856	0.798	0.753	0.561
cross validate	0.874	0.868	0.855	0.788	0.912	0.910	0.844	0.793	0.786	0.562
Indelocator	0.202	0.110	0.048	0.023	0.729	0.382	0.064	0.017	0.001	0.000
VarScan	0.218	0.211	0.124	0.115	0.390	0.390	0.184	0.182	0.020	0.018
VarDict	0.707	0.635	0.619	0.525	0.408	0.319	0.393	0.296	0.311	0.165
	Recall									
0	0.847	0.791	0.762	0.667	0.979	0.976	0.871	0.764	0.765	0.497
10	0.732	0.673	0.658	0.562	0.625	0.593	0.529	0.443	0.360	0.158
20	0.754	0.687	0.693	0.573	0.695	0.664	0.595	0.441	0.412	0.277
50	0.786	0.721	0.727	0.605	0.773	0.739	0.668	0.547	0.491	0.277
100	0.801	0.746	0.736	0.620	0.796	0.771	0.684	0.588	0.516	0.324
200	0.809	0.760	0.739	0.638	0.807	0.811	0.709	0.623	0.571	0.353
500	0.812	0.768	0.744	0.644	0.828	0.829	0.739	0.649	0.601	0.392
1000	0.818	0.772	0.747	0.650	0.864	0.855	0.765	0.673	0.626	0.397
cross validate	0.807	0.776	0.754	0.656	0.864	0.864	0.747	0.668	0.688	0.399
	Precision									
0	0.212	0.201	0.195	0.175	0.084	0.084	0.075	0.067	0.067	0.044
10	0.822	0.824	0.866	0.834	0.828	0.788	0.826	0.848	0.734	0.788
20	0.895	0.904	0.908	0.893	0.881	0.857	0.890	0.912	0.841	0.857
50	0.915	0.928	0.940	0.924	0.898	0.895	0.931	0.927	0.907	0.910
100	0.927	0.947	0.956	0.950	0.909	0.913	0.946	0.953	0.916	0.927
200	0.936	0.955	0.969	0.953	0.928	0.913	0.945	0.956	0.928	0.934
500	0.944	0.969	0.974	0.973	0.952	0.954	0.957	0.967	0.935	0.946
1000	0.945	0.977	0.980	0.980	0.964	0.963	0.972	0.979	0.945	0.956
cross validate	0.952	0.985	0.986	0.985	0.966	0.961	0.971	0.940	0.917	0.951
T.P. fraction	0.212	0.201	0.195	0.175	0.084	0.084	0.075	0.067	0.067	0.044

Table 19. Reduced size of training data set for SomaticSeq INDELs. Size is defined by the number of true positives. The final row (T.P. fraction) is the fraction of true positives in the training data for each data set, i.e., for DC3A (DREAM Challenge Stage 3, Setting A), the fraction of true positives is 0.138. Thus, when there are 10 true positives, the total training data consisted of $10/0.212 = 47$ calls (of which 10 are true positives and 37 are false positives). 0 means no SomaticSeq training. F_1 score of the individual tools are included for easy comparison.

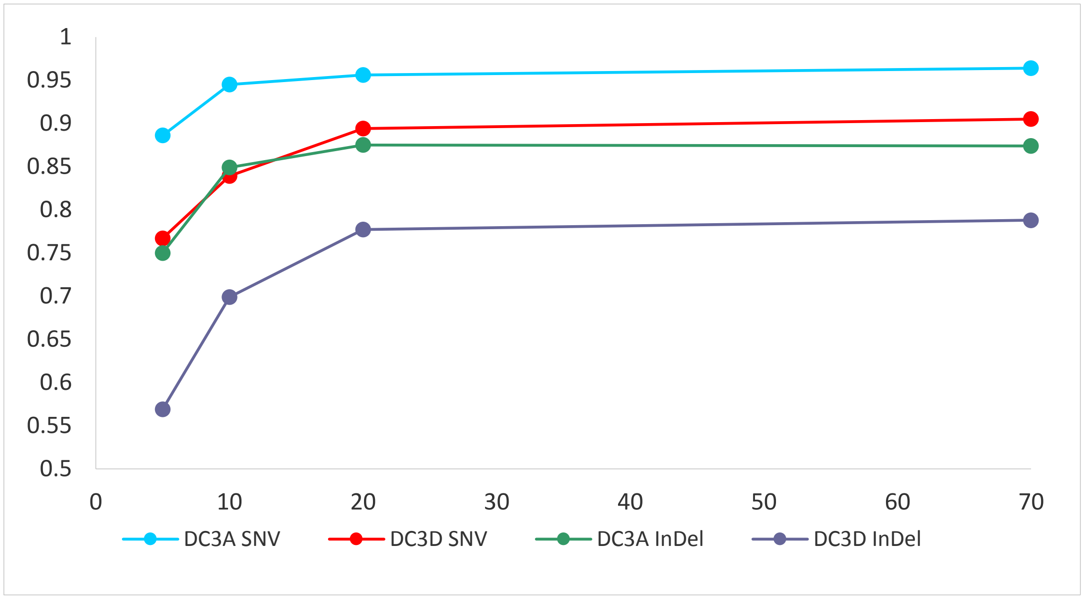

FIGURE 2. The F_1 scores vs. number of features. The gain in accuracy diminishes after around top 20 features with the most predictive values. The detailed data are presented in Table 17.

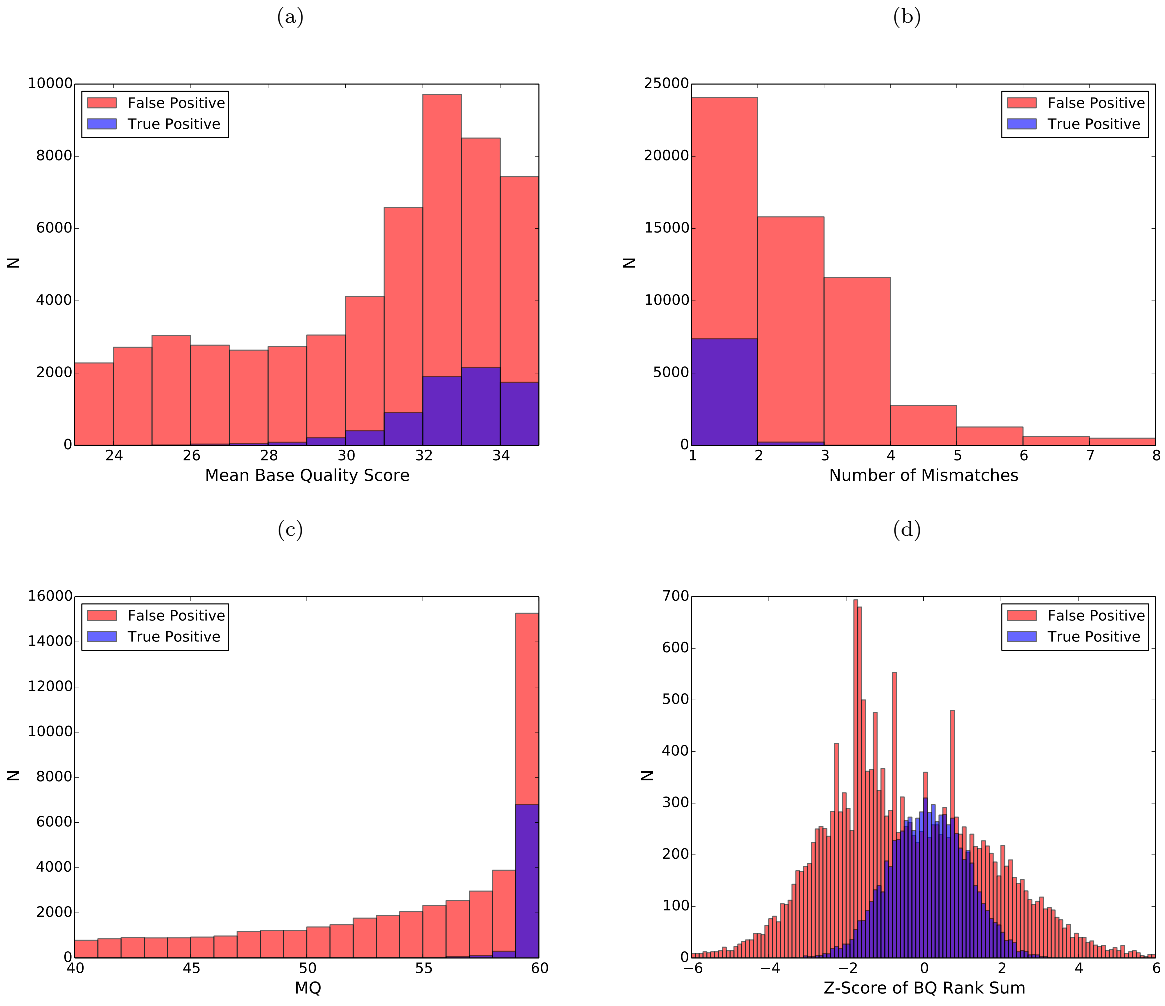

Figure 3. (a) Mean base quality score in tumor is often the most important feature in the machine learned classifier. Sequencing errors occur more frequently than somatic mutations. Thus, a variant call based on poor base quality is more likely a sequencing error than a true somatic mutation. (b) Number of mismatches reported by VarDict. The vast majority of the true somatic mutations have one mismatch in the read: the variant base itself. (c) Root-mean-square mapping quality (MQ) score of the tumor reads. MQ is a strong predictor, showing that almost all true somatic mutations have MQ above 57. (d) z-score of base quality rank sum between the reference and alternate reads in tumor reads. It is a measure of base quality bias between the reference and alternate reads. This is a weaker predictor than BQ, but also holds value as large-magnitude z-scores are enriched with false positives comparing to z-scores close to 0. All figures here are generated from Stage 3 of DREAM Challenge data.

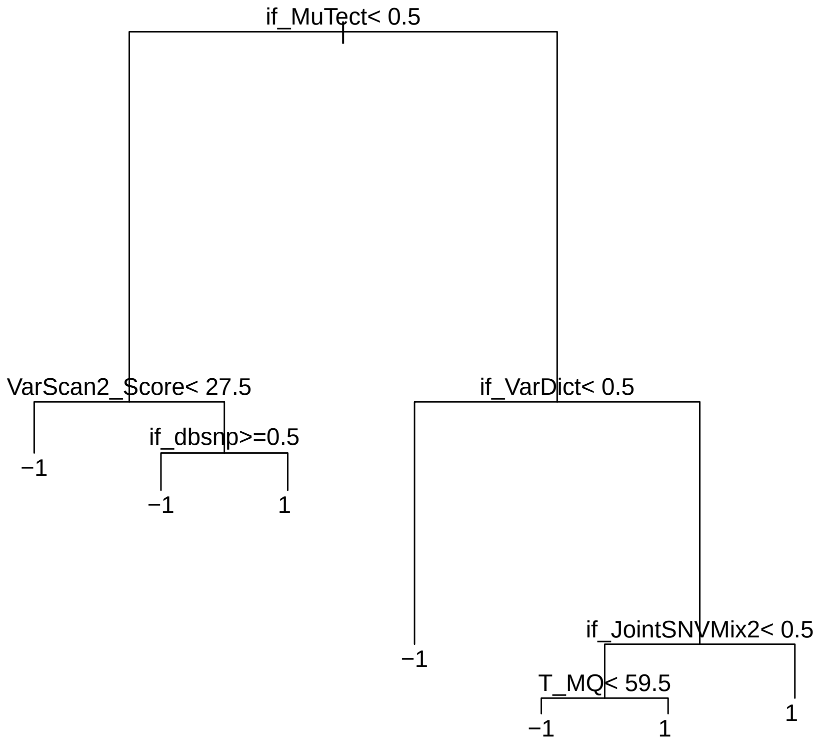

FIGURE 4. The first of 1000 decision trees from the combined data set of DREAM Settings A and B. T_MQ denotes the root mean square mapping quality score of the tumor.

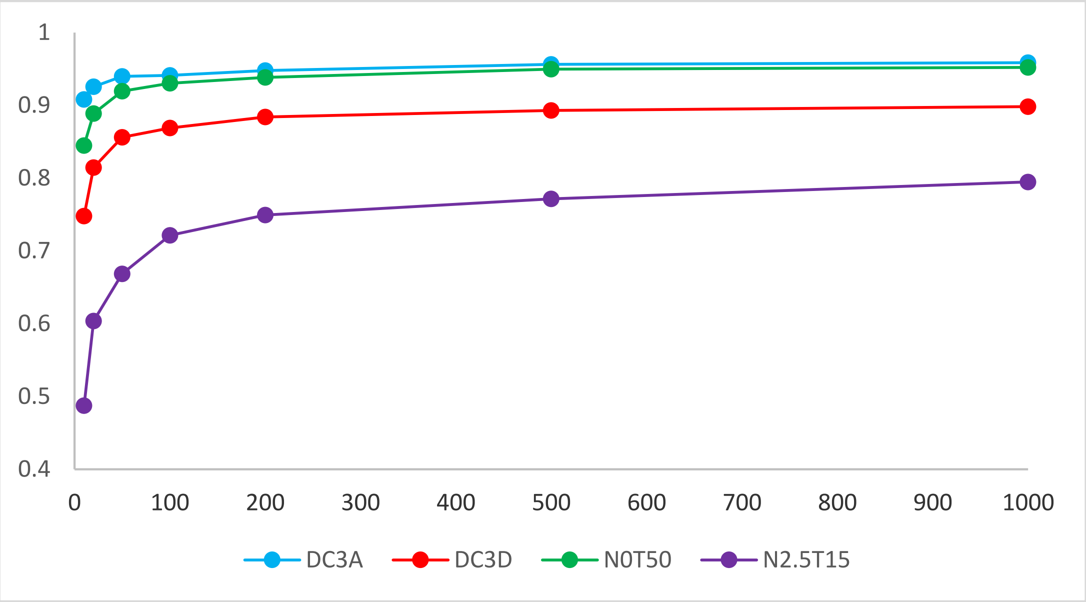

FIGURE 5. The F_1 scores vs. size of the training data set (number of true positives). The gain in accuracy diminishes as the size increases. The detailed data are presented in Table 18. For comparison, the best individual tool's F_1 scores were 0.789 (MuTect), 0.624 (MuTect), 0.607 (SomaticSniper) and 0.296 (VarDict) for DC3A, DC3D, N_0T_{50}, and $N_{2.5}T_{15}$, respectively.

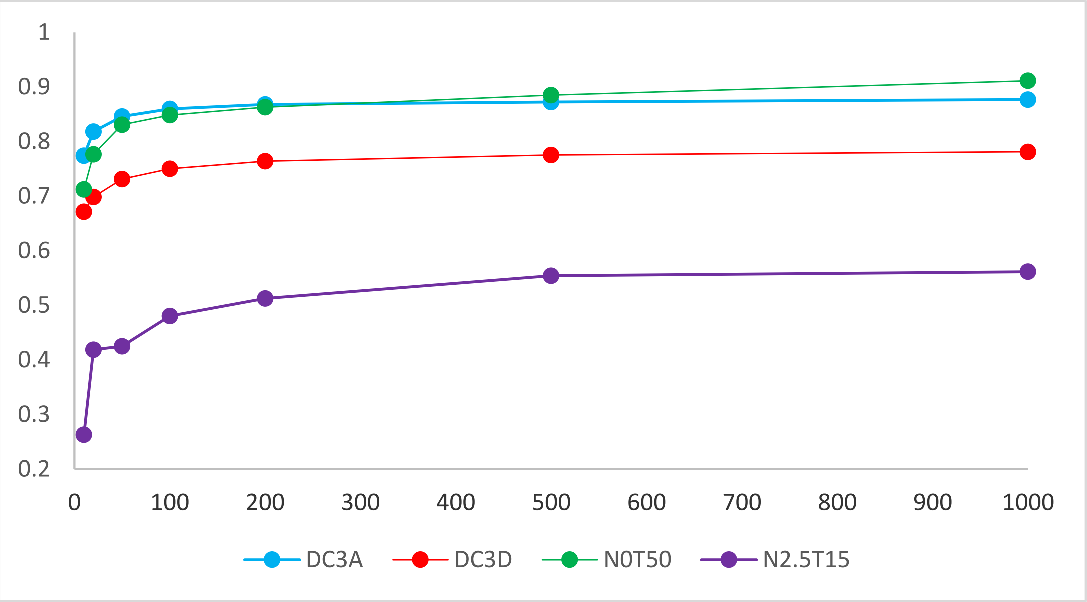

FIGURE 6. The F_1 scores vs. size of the training data set (number of true positives) for INDELs. The gain in accuracy diminishes as the size increases. The detailed data are presented in Table 19. For comparison, the best individual tool's F_1 scores were 0.707 (VarDict), 0.525 (VarDict), 0.729 (Indelocator), and 0.165 (VarDict) for DC3A, DC3D, N_0T_{50}, and $N_{2.5}T_{15}$, respectively.

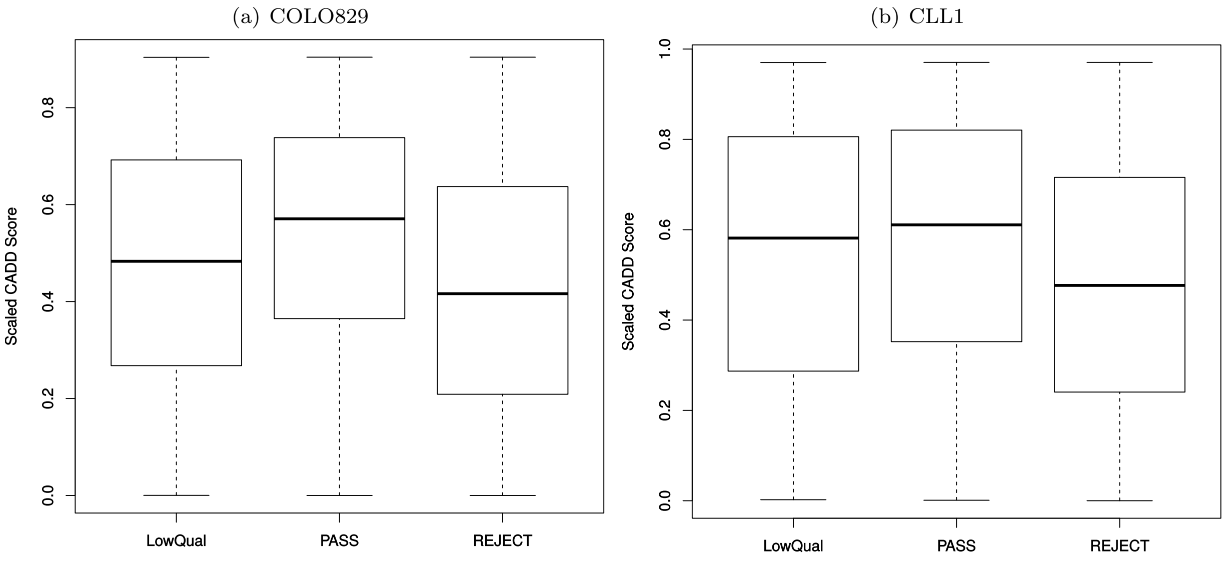

FIGURE 7. CADD rankscores for all SNVs reported by SomaticSeq for (a) COLO829 and (b) CLL1.
